# KLF5 protects the intestinal epithelium against Th17 immune response in a murine colitis model

**DOI:** 10.1172/jci.insight.153488

**Published:** 2022-04-08

**Authors:** Jason Shieh, Timothy H. Chu, Yang Liu, Julie Kim, Ainara Ruiz de Sabando, Soma Kobayashi, Sui Y. Zee, Brian S. Sheridan, Agnieszka B. Bialkowska, Vincent W. Yang

**Affiliations:** 1 Department of Medicine,; 2 Department of Microbiology and Immunology,; 3 Department of Biomedical Informatics,; 4 Department of Pathology, and; 5 Department of Physiology and Biophysics, Renaissance School of Medicine at Stony Brook University, Stony Brook, New York, USA.

**Keywords:** Gastroenterology, Inflammation, Cytokines, Inflammatory bowel disease, Mouse models

## Abstract

Inflammatory bowel disease (IBD) is a chronic illness characterized by dysregulated immune cascades in the intestines, in which the Th17 immune response plays an important role. We demonstrated that mice with intestinal epithelium–specific deletion of *Krüppel-like factor 5* (*Klf5*) developed Th17-dependent colonic inflammation. In the absence of KLF5, there was aberrant cellular localization of phosphorylated STAT3, an essential mediator of the Th17-associated cytokine, IL-22, which is required for epithelial tissue regeneration. In contrast, mitigation of IL-17A with anti–IL-17A neutralizing antibody attenuated colitis in *Klf5*-deficient mice. There was also a considerable shift in the colonic microbiota of *Klf5*-deficient mice that phenocopied human IBD. Notably, the inflammatory response due to *Klf5* deletion was alleviated by antibiotic treatment, implicating the role of microbiota in pathogenesis. Finally, human colitic tissues had reduced KLF5 levels when compared with healthy tissues. Together, these findings demonstrated the importance of KLF5 in protecting the intestinal epithelium against Th17-mediated immune and inflammatory responses. The mice described herein may serve as a potential model for human IBD.

## Graphical abstract

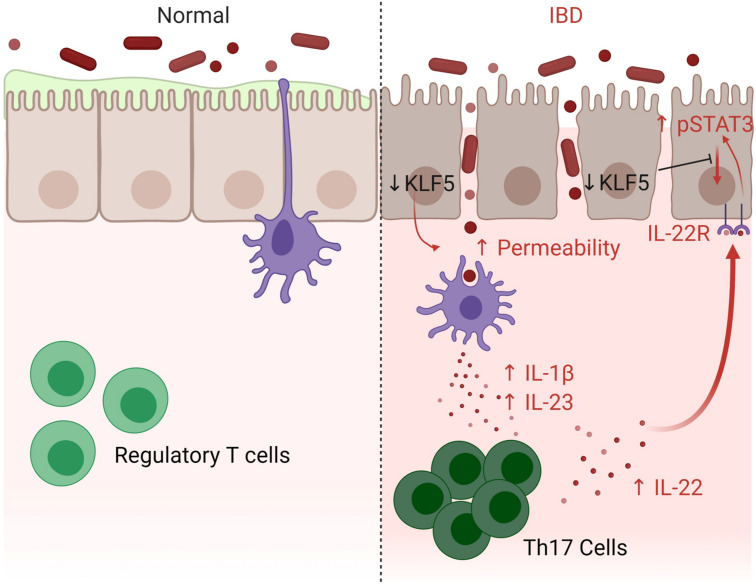

## Introduction

Inflammatory bowel disease (IBD) is a chronic inflammatory condition of the intestines driven in part by an imbalanced intestinal microenvironment. IBD comprises 2 major subtypes: ulcerative colitis (UC) and Crohn’s disease (CD) (1). Although both subtypes are classified under the same disease, they have distinct clinical features and anatomical distribution. UC is confined to the colon and can affect regions from the anorectal margin to proximal colonic regions as a continuous patch (1). On the other hand, CD can occur in any part of the gastrointestinal tract, most commonly the terminal ileum or right-sided colon, and often has perianal involvement. CD has the typical appearance of “patchy lesions” on colonoscopy and, if present, granulomas on pathology. Clinically, patients with CD often experience weight loss and, not infrequently, abscesses or fistula formation (1). Despite the differences between these 2 subtypes, they both manifest dysregulated immune and inflammatory responses that ultimately disrupt intestinal homeostasis.

Several key elements are involved in maintaining intestinal epithelial homeostasis, including intestinal epithelial cells, stromal cells with associated mucosal immune cells, and the microbiota (2). The intestinal microbiota is typically composed of bacteria beneficial for gut homeostasis and, therefore, the host (3). In healthy tissue, the intestinal epithelium maintains a barrier to limit the mucosal invasion by luminal microbiota. This barrier has 2 layers: a physical one upheld by epithelial tight junctions and a chemical one composed of molecules from the regenerating protein family and mucin glycoprotein family (4–6). The immune system helps shape the luminal niche within the mucosal tissue by sampling microbiota and eliciting inflammation to pathogenic bacterial populations (7–9). In dysbiosis, a condition in which the gut bacteria become unbalanced and their benefits to the host become subverted, a proinflammatory response is initiated to limit bacterial distribution through autophagy (10, 11), immune clearance of pathogens, and repair signals for intestinal epithelial cells (12). As such, a delicate balance to maintain the intestinal epithelial barrier, microbiota, and inflammatory response is essential for a healthy gut.

Given that Th17 responses are strongly correlated with inflamed regions of the intestine of patients with IBD (13), it is imperative to understand how these cells shift the homeostatic balance among the intestinal epithelial barrier, microbiota, and other components of the immune system. The major cytokine associated with Th17 is IL-17A; however, secukinumab, a mAb against IL-17, paradoxically exacerbates gastrointestinal symptoms in patients with IBD (14). As such, the focus has shifted to other cytokines that Th17 produces, such as IL-22. IL-22 is of particular interest because it functions to promote the regeneration of intestinal epithelial cells (13). RORγT, a hallmark transcription factor that controls the production of IL-22, is highly expressed in Th17, γδ T cells, and type 3 innate lymphoid cells (15–18). IL-22 is an essential effector cytokine that regulates the intestinal epithelial response by signaling through its heterodimeric receptor, IL-22R, composed of IL-22R1 and IL-10Rβ. The IL-22 receptor complex then signals through phosphorylation of STAT3 (19). Members of the STAT family typically reside in the cytosol but are activated and translocated into the nucleus upon phosphorylation. IL-22R/STAT3 signaling promotes progression of the cell cycle, production of antiapoptotic factors, and increase in mitogenic factors (20). Notably, the level of phosphorylated STAT3 (p-STAT3) in colonic tissues of patients with IBD is higher when compared with that in healthy individuals, suggesting cytokines that signal through STAT3 may be of importance (21, 22). IL-22 plays a prominent role in restoring homeostasis through p-STAT3 by restoring the mucosal layer through epithelial cell regeneration; however, the exact mechanism of aberrant regeneration and increased p-STAT3 in patients with IBD requires further elucidation.

Several IBD models exist but are limited in their ability to capture the interaction among the intestinal epithelial barrier, immune response, and microbiota. Here, we characterized a mouse model with inducible deletion of Krüppel-like factor 5 (KLF5) from the intestinal epithelium, which we showed to accurately recapitulate the immune response and pathological features of IBD in patients.

KLF5 is a pro-proliferative zinc finger transcription factor with essential roles in morphogenesis, wound healing, and proliferation, among other critical biological processes (23–25). Previous work has also shed light on the crucial role of KLF5 in maintaining the intestinal stem cell populations in the intestinal epithelium (26). Intestinal stem cell signature genes, such as *Lgr5*, *Olfm4*, *Ascl2*, and *Smoc2*, were decreased upon *Klf5* deletion. Furthermore, we demonstrated an impact of *Klf5* deletion on distribution of intestinal epithelial cell lineages, notably a decrease in secretory lineages and an increase in enterocyte populations (27). In addition, absence of KLF5 from the intestinal epithelium attenuates epithelial barrier integrity by decreasing desmosome complex formation (24, 28). Importantly, heterozygous *Klf5*-KO mice are more susceptible to dextran sulfate sodium–induced (DSS-induced) colitis than WT mice (23). Consistently, WT mice have an increased level of KLF5 in the crypt epithelium upon DSS treatment, suggesting that KLF5 is important in guarding against DSS-induced colitis. Coupled with KLF5’s role in maintaining epithelial barrier integrity and stem cell function, mice with inducible intestinal epithelium–specific *Klf5* ablation can serve as a model for human IBD.

## Results

### KLF5 is reduced in the colon of patients with active colitis.

**Figure 1:**
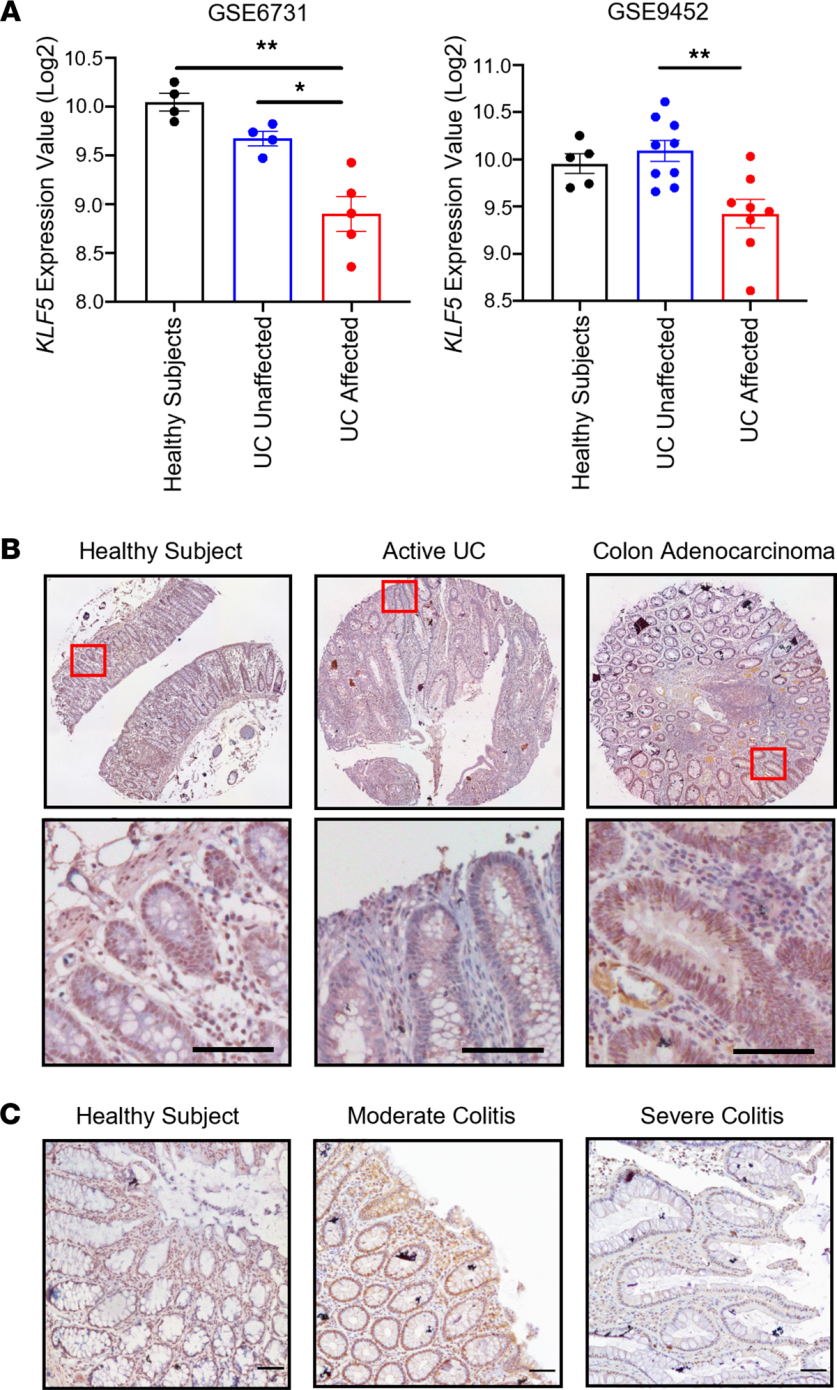
Patients with severe colitis have decreased levels of KLF5 in colonic epithelial cells. (**A**) Quantification of KLF5 expression value from 2 independent UC patient data sets. Samples include healthy individuals, UC-unaffected regions, and UC-affected regions. (**B**) IHC staining of KLF5 in human colon biopsy tissue array. The left is healthy tissue, the middle is active UC tissue, and the right is colon adenocarcinoma tissue. (**C**) IHC staining of KLF5 in human colon biopsies from Stony Brook University Biobank. The left is healthy colon tissue, the middle is moderate colitis tissue, and the right is severe colitis tissue. Data from graphs represent mean ± SEM, *P < 0.05; **P < 0.01; 1-way ANOVA. Scale bars: 70 μm.

We first evaluated the role of KLF5 in the pathogenesis of human colitis by measuring its levels in patient samples. Meta-analysis of DNA microarray data from 2 independent National Center for Biotechnology Information (NCBI) Gene Expression Omnibus (GEO) data sets showed significant decreases of *KLF5* mRNA levels in inflamed (affected) regions of UC samples when compared with either unaffected regions or healthy individuals (Figure 1A) (29). We then performed IHC staining of KLF5 on arrays containing patient tissues. Samples from healthy individuals and colon adenocarcinoma biopsies were used as controls, and both had high levels of KLF5 (Figure 1B). In comparison with healthy individual and colon adenocarcinoma samples, samples of patients with active UC had a substantial decrease in KLF5 level in colonic epithelial cells (Figure 1B); moderate colitis had decreased KLF5 expression compared with healthy individuals, but this reduction was more pronounced in areas of severe colitis than moderate colitis or healthy individuals (Figure 1C). The correlation between reduced KLF5 levels and colitis suggested that KLF5 may play a pathogenic role, prompting us to examine the mouse intestine after KLF5 ablation.

### Mice with intestinal epithelium–specific deletion of Klf5 develop colitis and have decreased survival.

**Figure 2:**
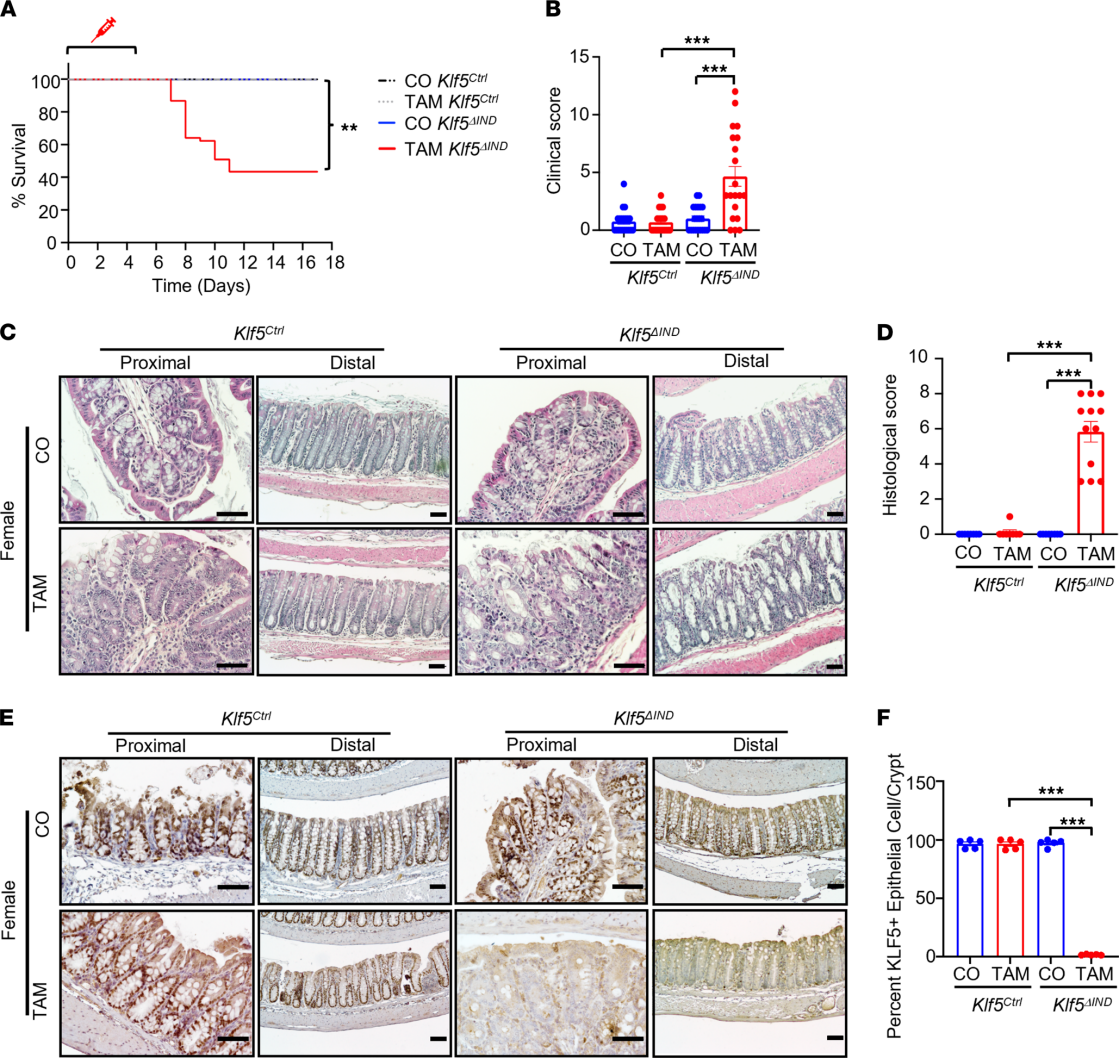
Deletion of *Klf5* from intestinal epithelial cells increases mortality and alters colonic morphology. (**A**) Kaplan-Meier survival curve of CO and TAM treatment for *Klf5^Ctrl^* and *Klf5^ΔIND^* mouse genotypes (*n* = 15). (**B**) Quantification of clinical scores of CO- and TAM-treated *Klf5^Ctrl^* and *Klf5^ΔIND^* mice. Max for the clinical score is 12 and quantified with 3 categories: stool consistency, weight loss, and fecal blood (*n* = 20). (**C**) H&E staining of whole colon tissue from female *Klf5^Ctrl^* and *Klf5^ΔIND^* mice treated with CO or TAM (*n* = 12). (**D**) Quantification of histological scores of CO- and TAM-treated *Klf5^Ctrl^* and *Klf5^ΔIND^* mice. Max for the histological score is 11 and quantified with 3 categories: crypt damage, inflammatory cells in lamina propria, and ulcers (*n* = 20). (**E**) IHC staining of KLF5 in whole colon tissue from female *Klf5^Ctrl^* and *Klf5^ΔIND^* mice treated with CO or TAM (*n* = 5). (**F**) Quantification for percentage of KLF5^+^ cells per crypt (*n* = 5). Data from graphs represent mean ± SEM, **P < 0.01; ***P < 0.001; 1-way ANOVA. Scale bars: 70 μm.

*Villin-CreER^T2^ Klf5^fl/fl^* (*Klf5*^ΔIND^) and *Villin-CreER^T2^* (*Klf5^Ctrl^*) mice were injected with tamoxifen (TAM) or corn oil (CO) control for 5 consecutive days and followed up to 18 days. TAM-treated *Klf5*^ΔIND^ mice had significantly decreased survival and higher clinical scores when compared with TAM-treated *Klf5^Ctrl^* mice and their CO-treated counterparts (Figure 2, A and B, respectively). Of note, TAM-treated female *Klf5*^ΔIND^ mice had significantly higher mortality and higher clinical scores than TAM-treated male *Klf5*^ΔIND^ mice (Supplemental Figure 1, A and B, respectively; supplemental material available online with this article; https://doi.org/10.1172/jci.insight.153488DS1).

In addition to higher mortality and clinical scores, the morphological changes in the colon of TAM-treated female *Klf5*^ΔIND^ mice exhibited inflammatory features, such as crypt distortion, crypt dilation, ulceration, and leukocyte infiltration in the lamina propria (Figure 2C). TAM-treated male *Klf5*^ΔIND^ mice had similar but milder morphological changes compared with TAM-treated female *Klf5*^ΔIND^ mice (Supplemental Figure 1C). Additionally, the histological score of TAM-treated *Klf5*^ΔIND^ mice was also significantly higher than TAM-treated *Klf5^Ctrl^* mice and their respective CO-treated counterparts (Figure 2D). Differences in histological score between TAM-treated *Klf5*^ΔIND^ males and females were again noted (Supplemental Figure 1D). Similar to the colon, morphological changes were also noted in the small intestine, specifically ileum, of TAM-treated *Klf5*^ΔIND^ mice of both sexes (Supplemental Figure 1E). Another feature unique to the small intestine during severe inflammation is villus blunting (highlighted in red square), which was only observed in TAM-treated *Klf5*^ΔIND^ female mice (Supplemental Figure 1E).

The absence of KLF5 from the colon of TAM-treated *Klf5*^ΔIND^ mice was confirmed by IHC staining, showing that deletion of *Klf5* from the colon of female *Klf5*^ΔIND^ mice (Figure 2, E and F) was more complete than that of male *Klf5*^ΔIND^ mice (Supplemental Figure 2A) and supporting the differences in phenotypical findings between the 2 sexes. We attributed these differences to a difference in recombination efficiencies between the sexes. This was confirmed by the significant decrease in the number of Klf5 transcripts in TAM-treated female *Klf5*^ΔIND^ mice as compared with TAM-treated male *Klf5*^ΔIND^ mice (Supplemental Figure 2B). Moreover, PCR analysis of the level of recombination of *LoxP* sites indicated more efficient recombination in TAM-treated female than male *Klf5*^ΔIND^ mice (Supplemental Figure 2, C and D). Given the more severe phenotype and resemblance to human colitis in TAM-treated female *Klf5*^ΔIND^ mice, they were selected for further studies.

### Genetic ablation of Klf5 leads to activation of global inflammatory pathways in the colon.

**Figure 3:**
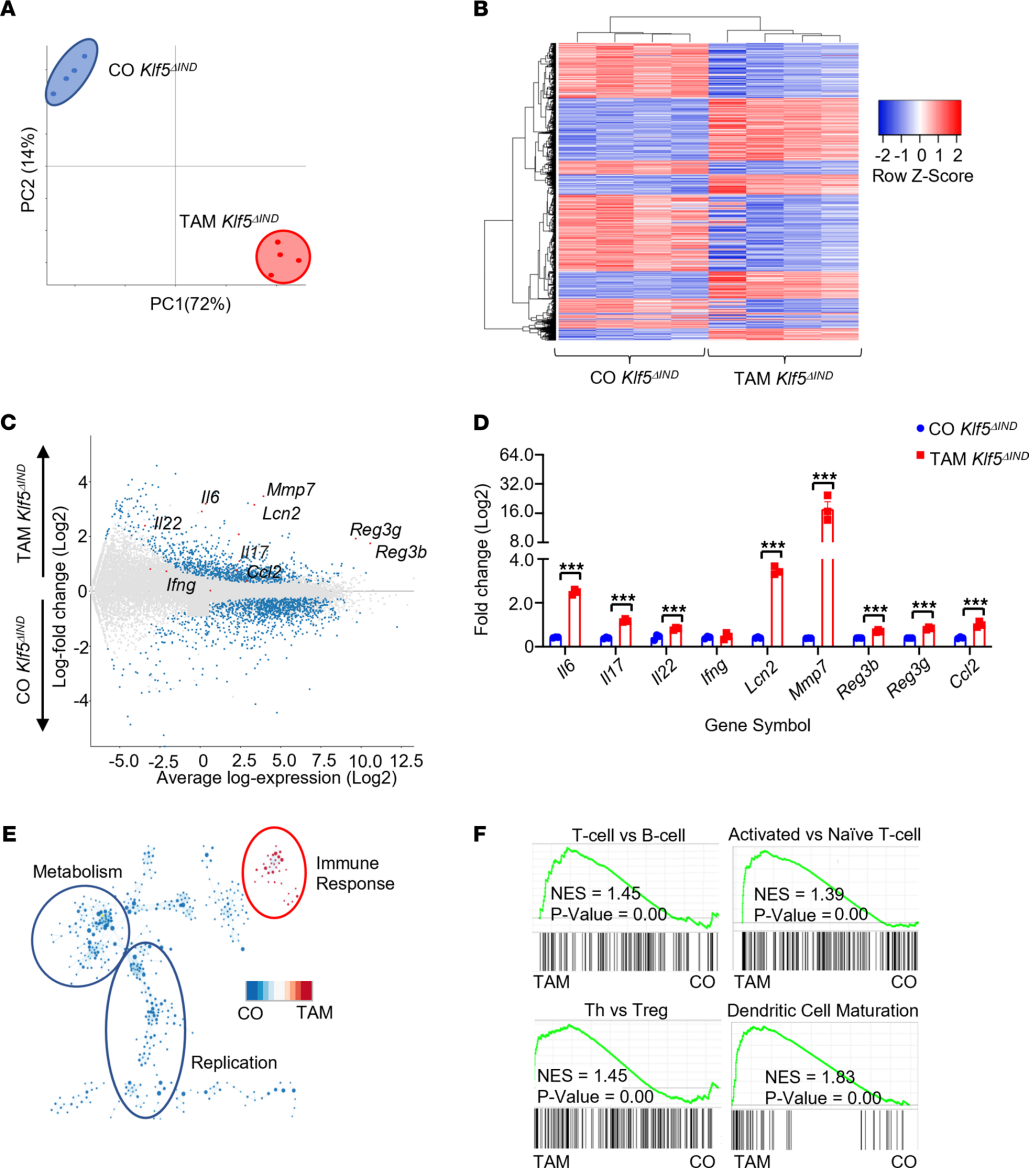
Deletion of *Klf5* from intestinal epithelial cells results in changes to the global transcriptomic pathways to inflammatory responses. (**A**) Principal component analysis of bulk RNA-Seq data. Comparison between CO- and TAM-treated *Klf5^ΔIND^* mice (*n* = 4). PC1 = 72% and PC2 = 14%. (**B**) Heatmap representation of significant differentially expressed genes from DESeq2. (**C**) MA-plot analysis of all genes from bulk RNA-Seq. Blue points represent a significant differentially expressed gene with a cutoff of *P* = 0.05. Red points are representative genes of interest with known roles in intestinal inflammation and regeneration. (**D**) RT-qPCR confirming genes of interest from bulk RNA-Seq analysis. (**E**) Leading-edge analysis after GSEA pathway analysis. Blue represents pathways correlated to CO-treated *Klf5^ΔIND^* mice and red represents pathways correlated with TAM-treated *Klf5^ΔIND^* mice. (**F**) GSEA pathway analysis positively correlated with TAM-treated *Klf5^ΔIND^* mice. Data from graphs represent mean ± SEM. ***P < 0.001; unpaired 1-tailed *t* test.

We examined the gene expression profiles in the mouse colon after *Klf5* deletion using RNA-Seq. The global transcriptomic landscape of TAM-treated *Klf5*^ΔIND^ mice was distinct from that of CO-treated *Klf5*^ΔIND^ mice based on principal component analysis and a hierarchically clustered heatmap (Figure 3, A and B, respectively). Highest-ranked genes after *Klf5* deletion belonged primarily to inflammatory pathways (Figure 3C). They included genes related to the inflammatory response (*Il17*, *Il22*, *Il6*, and *Ccl2*), intestinal translocation of bacteria (*Lcn2*, *Reg3b*, and *Reg3g*), and tissue regeneration and remodeling (*Mmp7*). Their expression levels were verified by quantitative reverse transcription PCR (RT-qPCR) (Figure 3D). In contrast, their levels were similar between *Klf5^Ctrl^* mice treated with CO or TAM (Supplemental Figure 3A). Importantly, we eliminated the possibility of off-target effects from CO or TAM treatment after comparing *Klf5^Ctrl^* mice with *Klf5*^ΔIND^ mice with identical treatments (Supplemental Figure 3, B and C).

Further analysis with gene set enrichment analysis (GSEA) and leading-edge analysis demonstrated that the highest-ranked pathways clustered and correlated with TAM-treated *Klf5*^ΔIND^ mice were associated with immune responses (Figure 3E) (30, 31). These pathways included activated T cell signaling, hallmark Th cell pathways, and DC maturation (Figure 3F). In contrast, pathways correlated with CO-treated *Klf5*^ΔIND^ mice CO were associated with metabolism and replication, such as oxidative phosphorylation, mitochondrial metabolism, and fatty acid catabolic process (Supplemental Figure 3D). Taken together, deletion of *Klf5* from the mouse colon resulted in a global transcriptomic shift toward strong inflammatory responses from Th cells.

### Deletion of Klf5 results in a Th17-mediated immune response in the colon.

**Figure 4:**
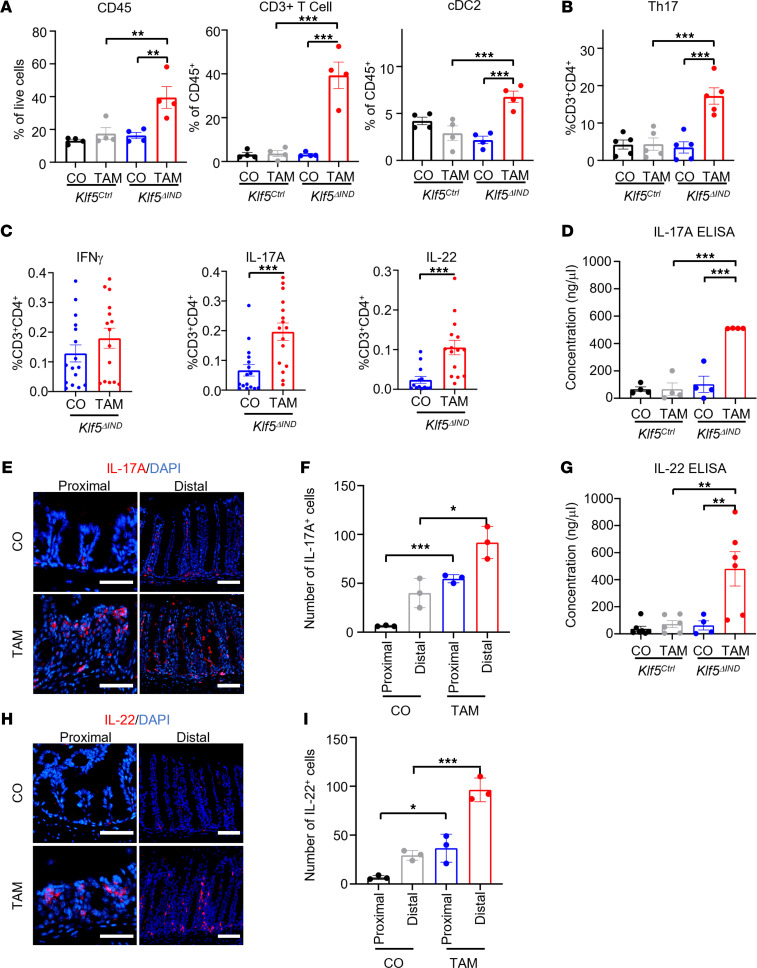
Intestine-specific deletion of *Klf5* results in increased Th17 cells and concentrations of IL-17 and IL-22. (**A**) Graph representation of gated populations. Graph on the left represents the percentage of CD45^+^ populations from the total number of live cells (*n* = 4). Middle graph represents the percentage of CD3^+^ cells out of the total CD45^+^ cells (*n* = 4). The right graph represents percentage of CD11c^+^ and CD103^+^ double-positive populations for conventional DC type 2 (cDC2) (*n* = 4). (**B**) Percentage of total CD4^+^ T cells that express the hallmark transcription factor for Th17, RORγT (*n* = 5). (**C**) Quantification of leukocytes treated with brefeldin A and ionomycin for cytokine stimulation. Percentages of cells with IFN-γ, IL-17A, and IL-22 were calculated from the total CD4^+^ T cell population (*n* = 16). (**D**) Quantification of IL-17A by ELISA from total tissue lysate (*n* = 4). (**E**) Immunofluorescence (IF) staining of IL-17A in whole colon tissue from CO- and TAM-treated *Klf5^ΔIND^* mice. (**F**) Quantification of cell numbers in proximal and distal colonic regions from the IF images (*n* = 3). (**G**) Quantification of IL-22 ELISA from total tissue lysate (*n* = 4). (**H**) IF staining of IL-22 in whole colon tissue from CO- and TAM-treated *Klf5^ΔIND^* mice. (**I**) Quantification of cell numbers in proximal and distal colonic regions from the IF images (*n* = 3). Data from graphs represent mean ± SEM, *P < 0.05; **P < 0.01; ***P < 0.001; 1-way ANOVA. Scale bars: 70 μm.

To identify the immune cells responsible for the global transcriptomic shift to an inflammatory state in the absence of *Klf5*, we performed flow analysis of pan-leukocyte populations from the lamina propria of CO- and TAM-treated *Klf5*^ΔIND^ mice (Supplemental Figure 4). There was a significant increase in the proportion of CD45^+^ cells in all acquired live cells from the colon of *Klf5*^ΔIND^ mice when compared with controls (Figure 4A). In addition, CD3^+^ T cells and conventional type 2 DCs (cDC2) were significantly increased (Figure 4A). By contrast, no other leukocyte subpopulations were significantly different between CO- and TAM-treated *Klf5*^ΔIND^ mice (Supplemental Figure 5, A–F). Finally, although there was not a significant difference in neutrophil populations between CO- and TAM-treated *Klf5*^ΔIND^ mice from flow analysis, we did note an increased presence of neutrophils in both the small intestine and colon of TAM-treated *Klf5*^ΔIND^ mice, particularly in areas containing crypt abscesses and cryptitis (Supplemental Figure 5J).

We then investigated the presence of Th cell transcriptional factors to distinguish among T cells: T-bet for Th1, GATA3 for Th2, and RORγT for Th17. A significant increase in Th17 cell population was observed in TAM-treated *Klf5*^ΔIND^ mice compared with TAM-treated *Klf5^Ctrl^* mice and CO-treated counterparts (Figure 4B). In contrast, there were no differences in the Th1 and Th2 cell population among all treatments and genotypes (Supplemental Figure 5, G and H, respectively). Additionally, we noted no difference in the type 3 innate lymphoid cell populations among the treatments and genotypes (Supplemental Figure 5I). To confirm the effect of *Klf5* deficiency in colonic epithelial cells on Th17 cell development, lymphocytes isolated from the colon were stimulated with PMA and ionomycin in the presence of brefeldin A, followed by measurement of IFN-γ, IL-17A, and IL-22 via intracellular cytokine staining. IL-17A and IL-22 are major effector cytokines, and IFN-γ is produced under exposure to specific pathogens. No difference was observed in IFN-γ–producing CD3^+^CD4^+^ cells between CO- and TAM-treated *Klf5*^ΔIND^ mice, which is consistent with the RT-qPCR and RNA-Seq data. By contrast, IL-17A and IL-22 were significantly increased in CD3^+^CD4^+^ cells from TAM-treated *Klf5*^ΔIND^ mice compared with those from CO-treated *Klf5*^ΔIND^ mice (Figure 4C). These results were further validated by ELISA and immunofluorescence (IF) staining of the affected tissues (Figure 4, D–I). Taken together, these results demonstrated that colitis in TAM-treated *Klf5*^ΔIND^ mice exhibited a strong Th17 phenotype that was associated with significant increases in IL-17A and IL-22 levels.

### Th-17–inducing factors are increased when Klf5 is deleted.

Since Th17 cells play a prominent role in the inflammatory response of TAM-treated *Klf5*^ΔIND^ mice, we measured the levels of factors that induce Th17 cells, including IL-1β, IL-23, and serum amyloid A. RNA-Seq analysis 5 days after TAM treatment did not reveal any significant difference in transcript numbers between CO- and TAM-treated *Klf5*^ΔIND^ mice for the 3 Th17 cell inducers (Supplemental Figure 6, A–C). However, 1 day after TAM treatment, there was a significant increase in the relative protein level of IL-1β and IL-23, but not that of SAA, by cytokine array (Supplemental Figure 6, D–F). Over a 7-day period of treatment, there was a significant increase in the concentration of IL-23 on days 1 and 3, but not later days, in tissues from TAM-treated mice compared with CO-treated mice (Supplemental Figure 6G). These results demonstrated that the levels of at least 2 of the Th-17–inducing factors were elevated relatively early in the course of TAM-induced deletion of *Klf5*.

### Neutralization of IL-17A by antibody attenuates colitis in Klf5-deficient mice.

**Figure 5:**
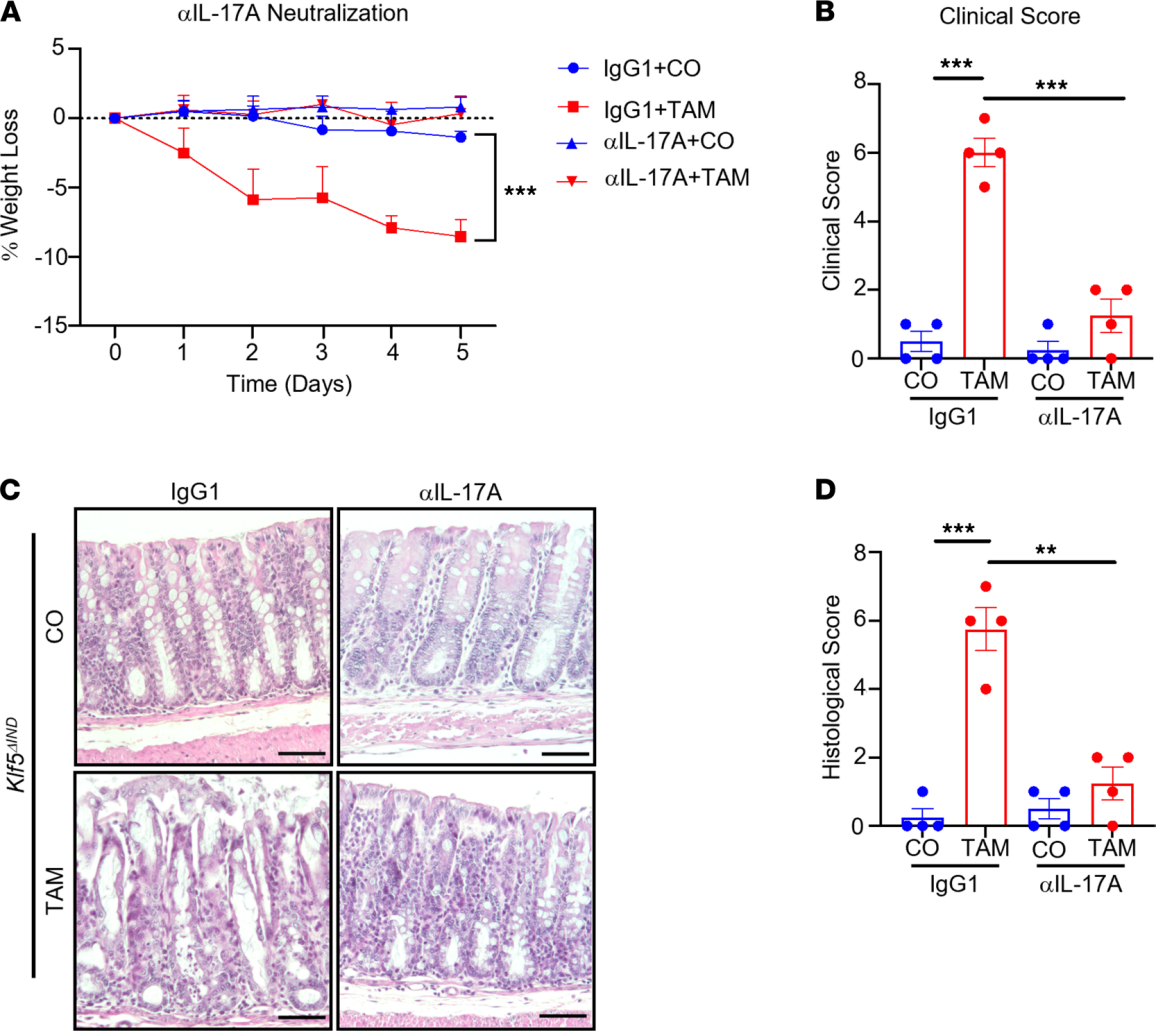
Th17 effector cytokines are essential for the pathogenesis of colitis when *Klf5* is deleted. (**A**) Weight change after 5 days of neutralizing antibody and CO or TAM treatment of *Klf5^ΔIND^* mice (*n* = 4). (**B**) Clinical scores. Max for the clinical score is 12 and quantified with 3 categories: stool consistency, weight loss, and fecal blood (*n* = 4). (**C**) H&E staining of *Klf5^ΔIND^* mice treated with IgG1 or αIL-17A neutralizing antibody and CO or TAM (*n* = 4). (**D**) Histological scores. Max for the histological score is 11 and quantified with 3 categories: crypt damage, inflammatory cells in lamina propria, and ulcers (*n* = 4). Data from graphs represent mean ± SEM. **P < 0.01; ***P < 0.001; 1-way ANOVA. Scale bar: 70 μm.

To understand the importance of Th17 effector cytokines in the pathogenesis of colitis, we administered anti–IL-17A (αIL-17A) neutralizing antibody or IgG1 control concurrently with CO or TAM for 5 consecutive days in *Klf5*^ΔIND^ mice. TAM-treated *Klf5*^ΔIND^ mice with IgG1 control had significant weight loss compared with CO-treated *Klf5*^ΔIND^ mice with IgG1 control (Figure 5A). In contrast, TAM-treated *Klf5*^ΔIND^ mice with αIL-17A neutralizing antibody did not lose weight compared to CO-treated *Klf5*^ΔIND^ mice with αIL-17A or IgG1 control (Figure 5A). Furthermore, only TAM-treated *Klf5*^ΔIND^ mice with IgG1 control had significantly increased clinical scores compared with the other groups (Figure 5B). Histologically, while TAM-treated *Klf5*^ΔIND^ mice with IgG1 control exhibited extensive morphological changes, such as crypt dilation, compared with CO-treated mice with either IgG1 control or αIL-17A, mice treated with TAM and αIL-17A had a more attenuated feature (Figure 5C). This was confirmed by histological scoring of the 4 groups of treatment combinations (Figure 5D). The finding that blocking IL-17A prevented development of colitis in TAM-treated *Klf5*^ΔIND^ mice implicates a causative role of IL-17A in the pathogenesis of colitis in these mice.

### Deletion of KLF5 results in aberrant cellular localization of p-STAT3 and defective STAT3 signaling.

**Figure 6:**
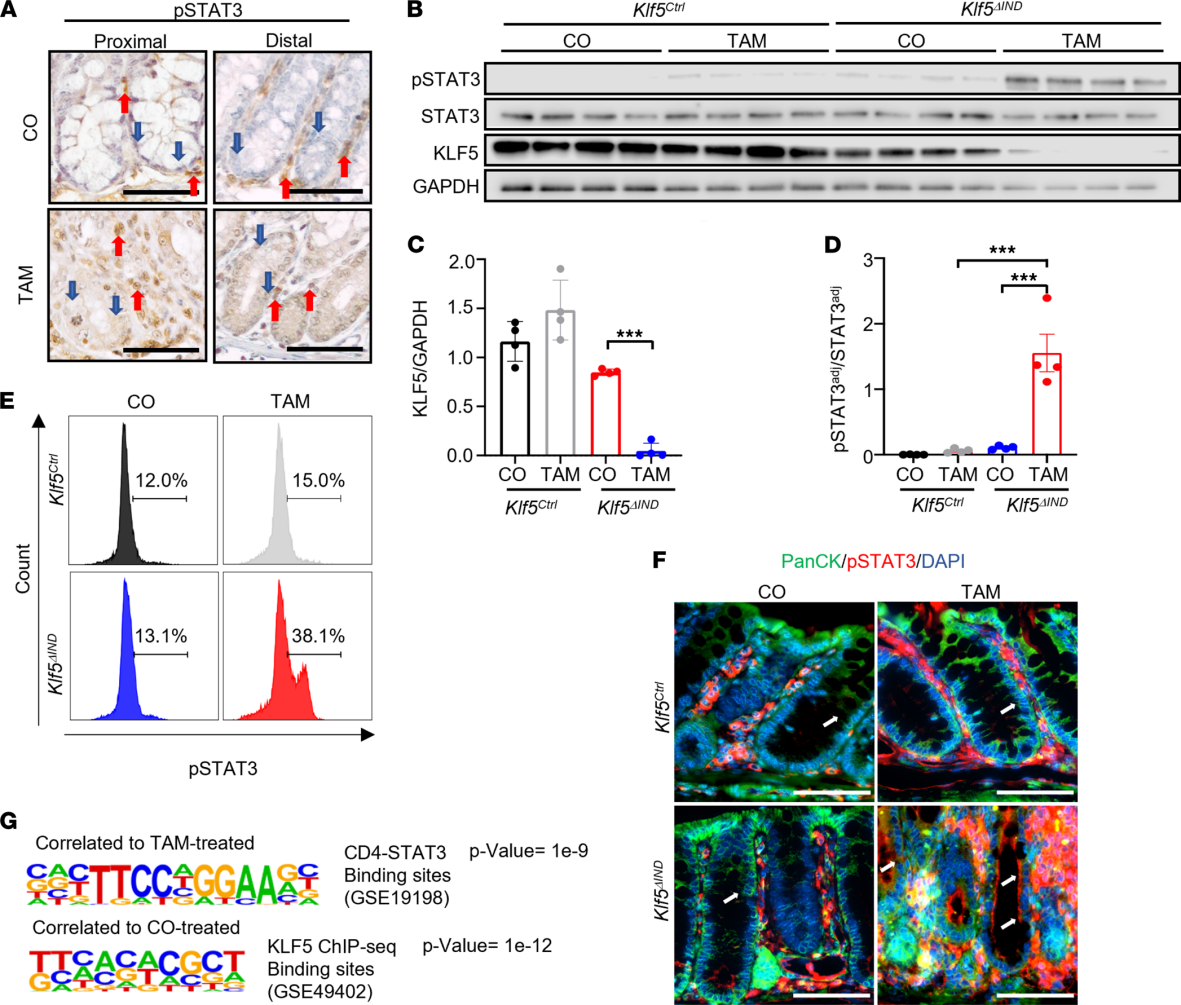
Deletion of *Klf5* results in increased p-STAT3 in intestinal epithelial cells but aberrant cellular localization. (**A**) IHC of p-STAT3 in CO- and TAM-treated *Klf5^ΔIND^* mice. Red arrows mark stromal regions of tissue. Blue arrows mark the intestinal epithelial layer (*n* = 5). (**B**) Western blot analysis of p-STAT3, STAT3, KLF5, and GAPDH from isolated epithelial cells (*n* = 4). (**C**) Quantification of KLF5 normalized with GAPDH. (**D**) Quantification of the adjusted p-STAT3 protein level over the adjusted STAT3 protein level. Adjustments were normalized with GAPDH for both markers (*n* = 4). (**E**) Histogram representation from FACS analysis of EpCAM^+^ cells that are gated p-STAT3^+^ (*n* = 6). (**F**) Immunofluorescence staining of colonic tissues with pan-cytokeratin and p-STAT3 antibodies. White arrows mark the intestinal epithelial layer (*n* = 4). (**G**) HOMER analysis of bulk RNA-Seq data (*n* = 4). The top motif represents the CD4-STAT3 binding sites, and the bottom motif presents the KLF5-binding sites. Data from graphs represent mean ± SEM. ***P < 0.001; 1-way ANOVA. Scale bars: 70 μm.

Results in Figure 4C show that in addition to IL-17A, levels of another Th17 cytokine, IL-22, were elevated in TAM-treated *Klf5*^ΔIND^ mice. Given IL-22’s function in inducing mucin production and epithelial regeneration (32), we examined the impact on its downstream signaling in epithelial cells upon *Klf5* deletion. Immunocytochemical analysis revealed increased p-STAT3 staining in TAM-treated *Klf5*^ΔIND^ mice compared with CO-treated *Klf5*^ΔIND^ mice (Figure 6A and Supplemental Figure 7A). These results were confirmed by Western blotting, FACS, IHC, and IF staining (Figure 6, B and D–F). Quantification of p-STAT3 IHC staining confirmed an increased percentage of p-STAT3^+^ epithelial cells per crypt (Supplemental Figure 7B). Deletion of *Klf5* was also confirmed with Western blots (Figure 6, B and C). When *Klf5* was present, p-STAT3 staining was largely confined to the lamina propria (Figure 6F and Supplemental Figure 7A). In contrast, upon *Klf5* deletion, p-STAT3 stained both lamina propria and epithelial cells, although p-STAT3 staining appeared to be localized primarily to the cytosol of epithelial cells by both IHC and IF staining (Figure 6, A and F, blue and white arrows, respectively).

To better understand whether aberrant cellular localization of p-STAT3 was epithelial cell autonomous or not cell autonomous (e.g., immune cells acting differently because of an altered cytokine environment), we analyzed the RNA-Seq data set using the HOMER motif scanner to determine common binding sites among differentially expressed genes (33). First, we confirmed that KLF5-binding sites were enriched in CO-treated and not in TAM-treated *Klf5*^ΔIND^ mice (Figure 6G). Next, increased p-STAT3–binding sites in TAM-treated *Klf5*^ΔIND^ mice were observed in CD4^+^ lymphocytes but not in epithelial cells (Figure 6G). We also determined the effect of *Klf5* on expression of *Il22ra1* and *Il22ra2*. By RNA-Seq and quantitative PCR, neither had any significant differences between CO-treated and TAM-treated *Klf5*^ΔIND^ mice (Supplemental Figure 7, C and D). These results suggest the aberrant cellular localization of p-STAT3 in the absence of KLF5 occurred in an epithelial cell–autonomous fashion.

**Figure 7:**
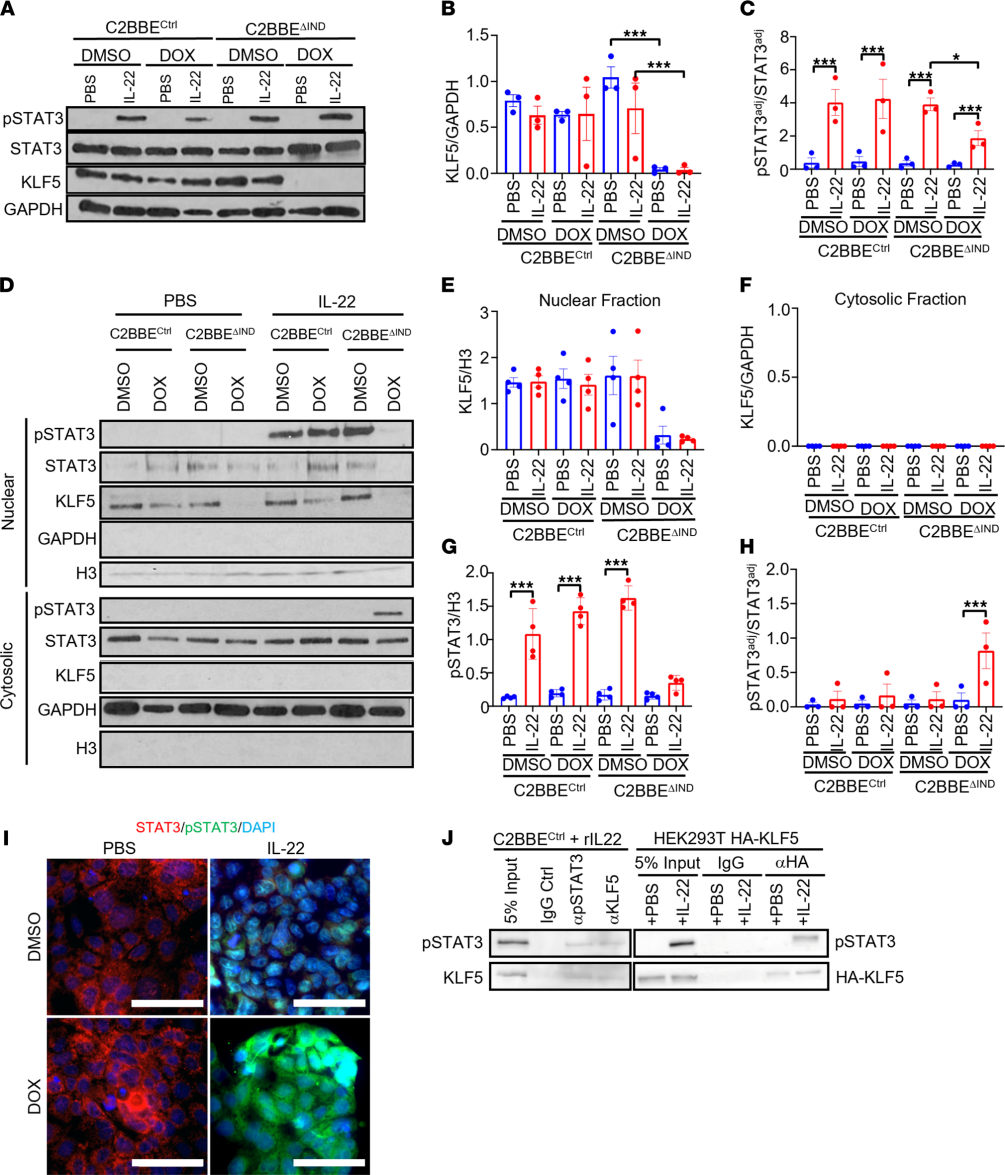
Knockdown of *KLF5* does not induce phosphorylation of STAT3 but affects the localization of p-STAT3 in response to IL-22 stimulation. (**A**) Western blots of C2BBE^Ctrl^ and C2BBE^ΔIND^ cells treated with a combination of DMSO or DOX and PBS or IL-22 (*n* = 3). (**B**) Quantification of KLF5 normalized with GAPDH (*n* = 3). (**C**) Quantifications of adjusted p-STAT3 protein level over the adjusted STAT3 protein level. Adjustments were normalized with GAPDH for both markers (*n* = 3). (**D**) Western blots of cytoplasmic-nuclear fractionation assays. The top blot represents the nuclear fractions and the bottom represents the cytosolic fractions. (**E** and **F**) Quantifications for KLF5 normalized to H3 in the nuclear fraction (**E**) and for KLF5 normalized to GAPDH in the cytosolic fraction (**F**) (*n* = 3). (**G**) Quantification of p-STAT3 protein normalized to H3 in the nuclear fraction (*n* = 3). (**H**) Quantification of p-STAT3 level adjusted to GAPDH to adjusted total STAT3 levels in the cytosolic fraction (*n* = 3). (**I**) STAT3, p-STAT3, and DAPI immunofluorescence staining of C2BBE^ΔIND^ cells treated with DMSO/DOX and PBS/IL-22. (**J**) Co-IP of p-STAT3 and endogenous (left panel) or overexpressed KLF5 (right panel). Data from graphs represent mean ± SEM. *P < 0.05; ***P < 0.001; 1-way ANOVA. Scale bars: 70 μm.

To further verify mislocalization of p-STAT3, a human adenocarcinoma colon cancer cell line, Caco-2BBE, with doxycycline-inducible (DOX-inducible) nonsilencing shRNA (C2BBE^Ctrl^) or DOX-inducible knockdown of *KLF5* (C2BBE^ΔIND^) was utilized (24). Caco-2BBE cells were treated with recombinant IL-22 (rIL-22) to examine the response of intestinal epithelial cells to IL-22. The results showed that IL-22 needed to be present in order to induce phosphorylation of STAT3 (Figure 7, A–C). Additionally, the level of p-STAT3 in response to IL-22 was lower in DOX-treated C2BBE^ΔIND^ than DMSO-treated C2BBE^ΔIND^ cells (Figure 7C). We then measured p-STAT3 levels in the nuclear and cytoplasmic fractions of PBS- and rIL-22–treated cells. Results in Figure 7D showed that while p-STAT3 exclusively resided in the nuclear fraction of cells with intact KLF5 after IL-22 treatment (Figure 7D; DMSO- and DOX-treated C2BBE^Ctrl^ and DMSO-treated C2BBE^ΔIND^), it was mostly present in the cytosolic fraction of cells in the absence of KLF5 after IL-22 treatment (Figure 7D; DOX-treated C2BBE^ΔIND^). The results of Western blots are quantified in Figure 7, E–H. The cytoplasmic localization of p-STAT3 in the absence of KLF5 after IL-22 stimulation was confirmed by IF staining (Figure 7I). These results indicate that KLF5 was necessary for IL-22–induced nuclear localization of p-STAT3.

Because we observed the mislocalization of p-STAT3 upon IL-22 stimulation in the absence of KLF5, we further investigated whether aberrant localization of p-STAT3 is exclusive to IL-22 signaling. IL-6 is a well-established activator of p-STAT3 through GP-130 and IL-6Rα. We administered recombinant IL-6 to C2BBE^ΔIND^ cells treated with DOX or DMSO and observed robust p-STAT3 nuclear localization in DMSO-treated C2BBE^ΔIND^ cells in response to IL-6 stimulation (Supplemental Figure 8; third column). On the contrary, p-STAT3 was largely localized to the cytoplasm of C2BBE^ΔIND^ cells treated with DOX and IL-6 (Supplemental Figure 8; fourth column). Taken together, these findings suggest that KLF5 has a ubiquitous role in modulating nuclear localization of p-STAT3 in response to different effectors.

To determine whether there is a physical interaction between KLF5 and p-STAT3, we performed co-IP experiments with KLF5 and p-STAT3 in C2BBE^Ctrl^ cells after rIL-22 stimulation. As shown in the left panel of Figure 7J, KLF5 was coprecipitated by a p-STAT3 antibody and vice versa. Moreover, p-STAT3 was coprecipitated by an HA antibody in HEK293T cells transfected with HA-tagged KLF5 followed by IL-22 treatment (Figure 7J). These results indicate that KLF5 assists in IL-22–induced nuclear localization of p-STAT3 by physically interacting with p-STAT3.

Finally, to understand the impact of aberrant p-STAT3 localization on IL-22 signaling due to KLF5 deletion, we measured the transcript numbers, by RNA-Seq, of known downstream targets of *Il22*, including *S100a8/9*, *Defb2*, and *Socs3*, in the colon of mice with or without *Klf5*. As shown in Supplemental Figure 7E, transcript numbers for each target were not significantly different between CO- and TAM-treated *Klf5*^ΔIND^ mice. These results confirmed that IL-22 signaling was defective in intestinal epithelial cells devoid of *Klf5*.

### Aberrant p-STAT3 localization in patients with UC.

After observing aberrant p-STAT3 staining in colonic epithelial cells of TAM-treated *Klf5*^ΔIND^ mice and DOX-treated C2BBE^ΔIND^ cells after IL-22 stimulation, we next investigated whether such a phenomenon is present in patients with colitis. IHC analysis of p-STAT3 showed negligible staining in normal healthy tissue and strong nuclear staining in the colon adenocarcinoma specimen (Supplemental Figure 9). In comparison, UC tissue had increased p-STAT3 staining compared with normal tissue but was diffusely present throughout the cells (Supplemental Figure 9). Combined with the observation that KLF5 was lower in active UC (Figure 1), results of these experiments implicate a role for KLF5 in modulating p-STAT3 signaling in human colitis as well.

### Deletion of KLF5 negates IL-22–induced proliferation.

**Figure 8:**
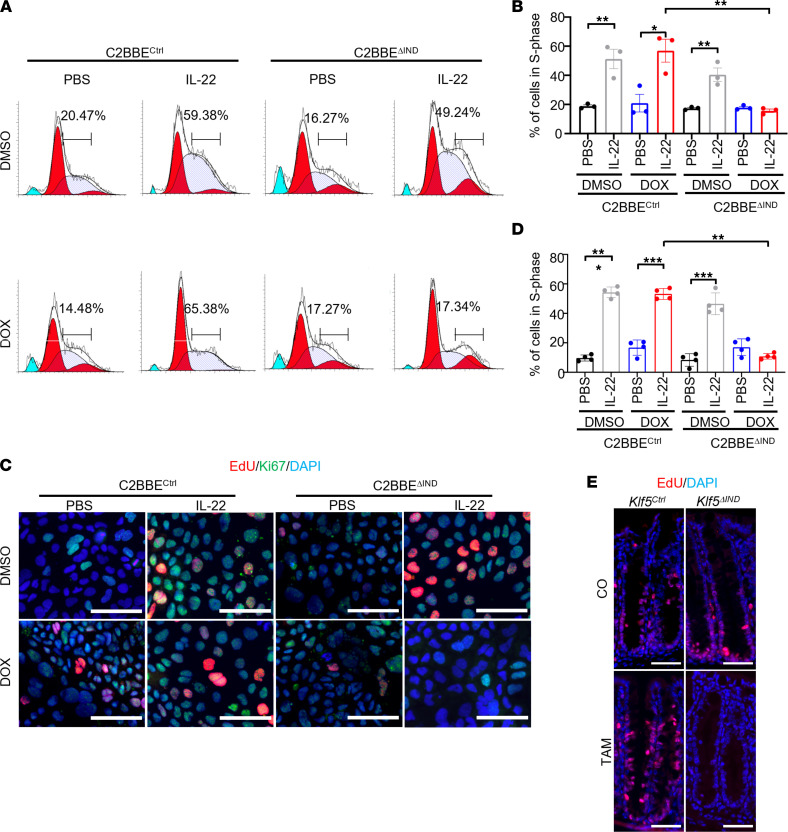
Knockdown of *KLF5* results in decreased proliferation in vitro and in vivo. (**A**) Cell cycling analysis histogram charts for ModFit. Quantifications on the chart represent the percentage of cells in the S-phase (*n* = 3). (**B**) Quantification of percentage cells in S-phase after ModFit curve fitting (*n* = 3). (**C**) Immunofluorescence (IF) staining of EdU and Ki67 in C2BBE^Ctrl^ and C2BBE^ΔIND^ cells treated with DMSO/DOX and PBS/IL-22. (**D**) Quantification of percentage of cells in S-phase from IF images (*n* = 3). (**E**) IF staining of EdU in CO- or TAM-treated *Klf5^Ctrl^* or *Klf5^ΔIND^* mice. Data from graphs represent mean ± SEM. *P < 0.05; **P < 0.01; ***P < 0.001; 1-way ANOVA. Scale bars: 70 μm.

As epithelial cell proliferation is an integral part of tissue regeneration after injury, we investigated the effects of *KLF5* deletion on IL-22–induced proliferative response. Cell cycling analysis showed that although rIL-22 significantly increased the proportion of cells in the S-phase of DMSO- or DOX-treated C2BBE^Ctrl^ cells or DMSO-treated C2BBE^ΔIND^ cells, it failed to do so when in DOX-treated KLF5^ΔIND^ cells (Figure 8, A and B). These results were confirmed by staining the cells for EdU and Ki67 (Figure 8, C and D). The aberrant signaling from IL-22 to p-STAT3 in the absence of KLF5 likely contributed to the absence of IL-22–stimulated proliferation in these cells. The lack of EdU incorporation in the intestinal epithelium in vivo was also observed in TAM-treated *Klf5*^ΔIND^ mice compared with TAM-treated *Klf5^Ctrl^* mice and CO-treated *Klf5^Ctrl^* or *Klf5*^ΔIND^ mice (Figure 8E).

### Deletion of Klf5 from colonoid culture results in reduced survival.

To understand the effects of *Klf5* deletion on intestinal epithelial differentiation, we performed ex vivo expansion of colonoids. Colonoids from *Klf5^Ctrl^* and *Klf5*^ΔIND^ mice were treated with 4-OHT, the metabolically active form of TAM, or PBS for 5 consecutive days. We confirmed that 4-OHT–treated *Klf5*^ΔIND^ colonoids resulted in decreased KLF5 expression compared with PBS-treated *Klf5*^ΔIND^ colonoids (Supplemental Figure 10, A and B). When *Klf5*^ΔIND^ colonoids were treated with 4-OHT, they had less budding compared with controls (Supplemental Figure 10, C and D). These findings showed that *Klf5* is essential for the maintenance of epithelial differentiation and expansion.

### IL-22 treatment of Klf5-deleted colonoids does not increase maintenance of budding and survival.

We observed perturbation of IL-22 signaling when *Klf5* was knocked down in C2BBE cells. To confirm these findings, we subjected colonoid cultures from *Klf5*^ΔIND^ mice treated with PBS or 4-OHT to stimulation with PBS or IL-22. The amount of budding and the size of colonoids were increased when *Klf5* was present from day 1 to day 5 of treatment (Supplemental Figure 11A, left 2 columns, and Supplemental Figure 11B). However, when *Klf5* was deleted, there was a decrease in budding colonoids regardless of whether the cells were treated with PBS or IL-22 (Supplemental Figure 11A, right 2 columns, and Supplemental Figure 11B). Altogether, the presence or absence of *Klf5* influenced the survival of colonoid cultures given that we observed decreased cellular proliferation when KLF5 was absent in C2BBE.

### Deletion of Klf5 results in altered microbial populations in the colon.

**Figure 9:**
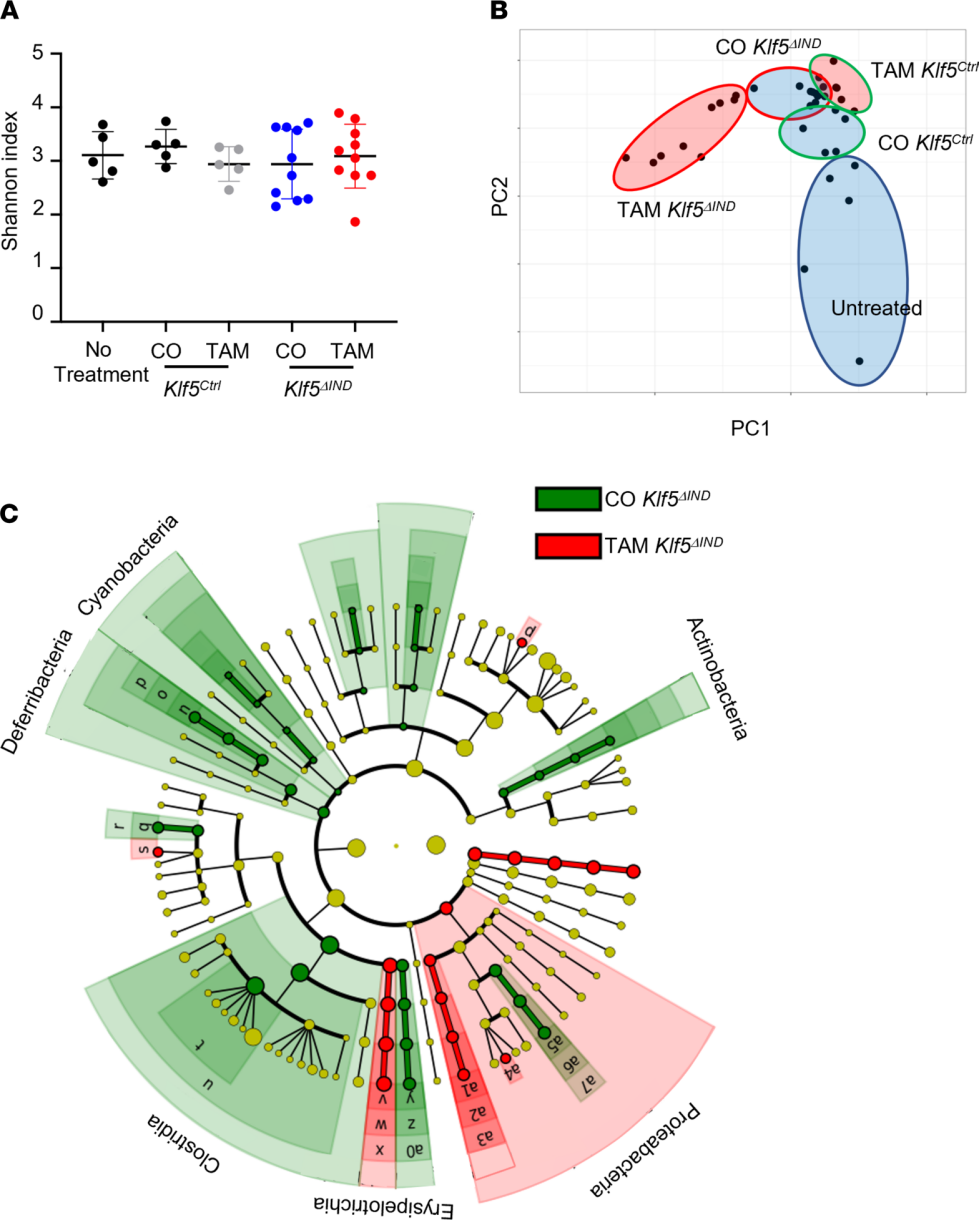
Microbiota from the colon of *Klf5*-deleted mice has no change in diversity but an increase in abundance. (**A**) Analysis of α diversity using the Shannon index. (**B**) Principal component analysis of 16s rRNA-Seq for *Klf5^Ctrl^* and *Klf5^ΔIND^* mice treated with CO or TAM. (**C**) Phylogenetic tree of linear discriminant analysis between CO- and TAM-treated *Klf5^ΔIND^* mice (*n* = 9).

To investigate the effects of *Klf5* deletion on the luminal microbiota, we performed 16s rRNA-Seq. Samples were collected 1 day after the last injection of CO or TAM. There were no changes in α diversity in the initial analysis using the Shannon index when the microbiota from Klf5-deleted mice was compared with that of control mice (Figure 9A). However, with principal component analysis, clusters were separated between CO and TAM treatment in *Klf5*^ΔIND^ mice, indicating changes in count number (Figure 9B). Linear discriminant analysis (LDA) showed significant phylogenetic order shifts between TAM- and CO-treated *Klf5*^ΔIND^ mice (Figure 9C and Supplemental Figure 12). Similar results were noted by comparing TAM treatment between *Klf5^Ctrl^* and *Klf5*^ΔIND^ mice (Supplemental Figure 13, A and B). Other permutations of comparison were performed to determine whether bulk changes in microbiota were exclusive to *Klf5* deletion, and there were minute differences between control mice (Supplemental Figure 13, C–F). Taken together, there were abundant changes in the microbiota composition in the absence of *Klf5*, suggesting that certain microbial populations may drive the inflammatory response as a consequence of *Klf5* deletion.

Segmented filamentous bacteria (SFB) and *Bacteroides* (BAT) are both essential microbes to maintain gut homeostasis. During colitis, though, SFB and BAT may contribute as pathobionts for Th17 cells. We performed quantitative PCR of the 16s rRNA sequence of SFB and BAT and observed a significant increase in transcript numbers for SFB and BAT in TAM-treated *Klf5*^ΔIND^ mice compared with CO-treated *Klf5*^ΔIND^ mice (Supplemental Figure 14, A and B). These results suggest that SFB, BAT, or both may play a role in the development of colitis after *Klf5* has been deleted.

### Antibiotic treatment abrogates Klf5 deletion–induced colitis.

**Figure 10:**
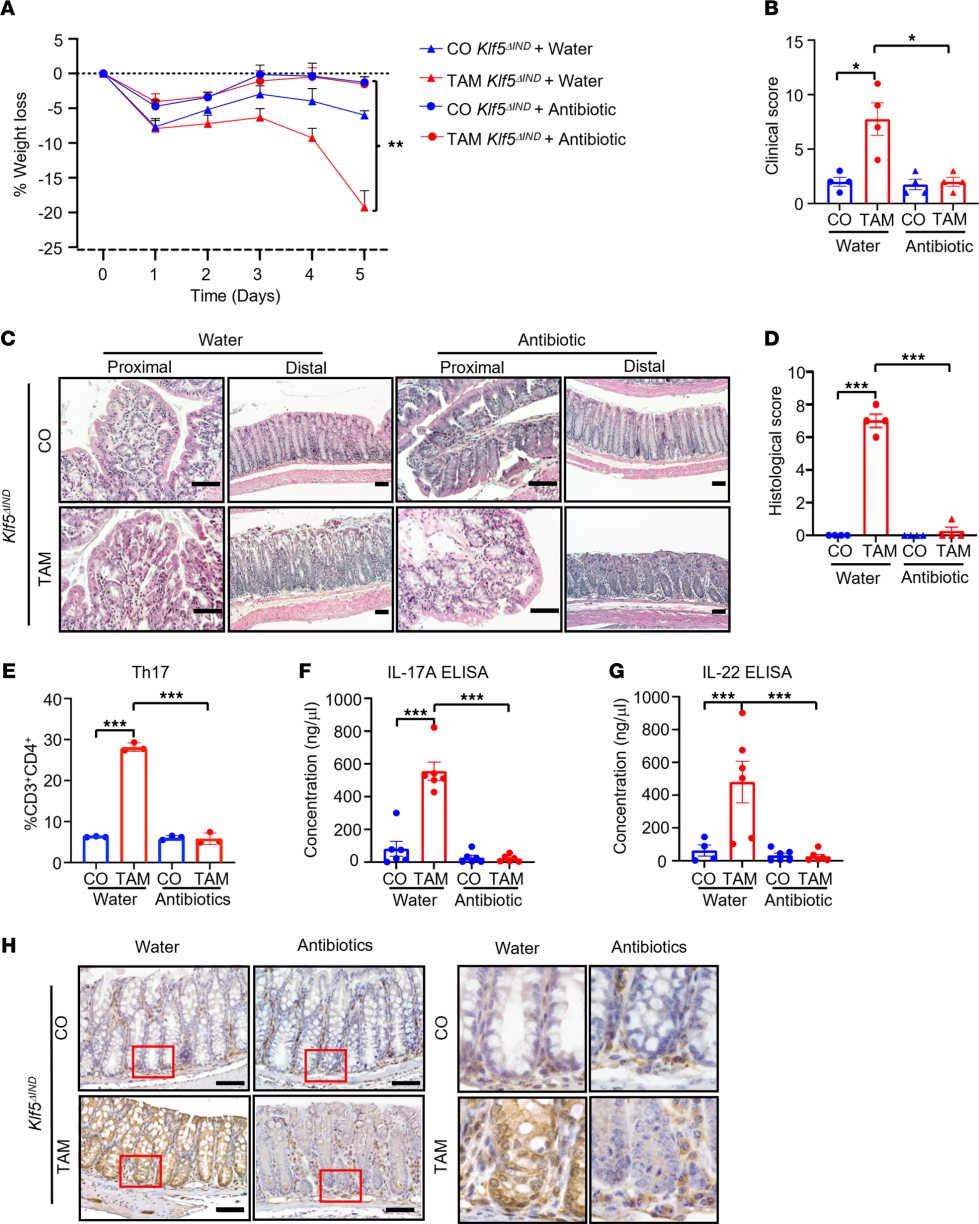
Colons of *Klf5*-deleted mice have reduced inflammation after antibiotic treatment. (**A**) The graph represents average weight change for CO- and TAM-treated *Klf5^ΔIND^* mice that also received water or antibiotic treatment (*n* = 3). (**B**) Clinical score quantification of CO- and TAM-treated *Klf5^ΔIND^* mice with water or antibiotic treatment (*n* = 4). (**C**) H&E staining of proximal and distal regions of the colon for water- or antibiotic-treated mice. (**D**) Histological score quantification of CO- and TAM-treated *Klf5^ΔIND^* mice with water or antibiotic treatment (*n* = 4). (**E**) The graph represents the percentage of total CD4^+^ T cells that express the hallmark transcription factor for Th17, RORγT (*n* = 3). (**F**) Quantification of IL-17A ELISA from total tissue lysates (*n* = 6). (**G**) Quantification of IL-22 ELISA from total tissue lysates (*n* = 4). (**H**) p-STAT3 IHC staining of distal colons from CO- and TAM-treated *Klf5^ΔIND^* mice with water or antibiotics. Data from graphs represent mean ± SEM. *P < 0.05; **P < 0.01; ***P < 0.001; 1-way ANOVA. Scale bars: 70 μm. Original magnification of insets, 20×.

Thus far, our data suggest that *Klf5* deletion from the intestinal epithelium leads to an inflammatory response and potentiates the development of colitis. Given that our previous work showed that deletion of *Klf5* is associated with reduced epithelial barrier integrity (24), we hypothesized that this compromised epithelial barrier with subsequent alteration of microbial flora is responsible for the inflammatory phenotype. Indeed, TAM-treated *Klf5*^ΔIND^ mice that were treated with an antibiotic cocktail had no significant weight reduction compared with CO-treated *Klf5*^ΔIND^ mice, which contrasts with the significant weight loss seen in TAM-treated *Klf5*^ΔIND^ mice given water only (Figure 10A and Supplemental Figure 15A). CO-treated *Klf5*^ΔIND^ mice given either water or the antibiotic cocktail also maintained their weights (Figure 10A and Supplemental Figure 15A). Consistent with the finding on weight loss, quantitative comparisons of clinical and histological scores demonstrated that antibiotic cocktail treatment rescued the colitis phenotype based on both phenotypic and morphological criteria (Figure 10, B–D). *Klf5* levels in the colon were reduced in both water- and antibiotic-treated *Klf5*^ΔIND^ mice given TAM as compared with those given CO (Supplemental Figure 15B).

We performed flow cytometry analysis to investigate whether the Th17 response continued in the absence of the luminal microbiota. When given water, TAM-treated *Klf5*^ΔIND^ mice had a significantly elevated Th17 response (Figure 10E), along with elevated IL-17A and IL-22 levels (Figure 10, F and G, respectively) compared with CO-treated mice. In contrast, these increases were ameliorated after administration of the antibiotic cocktail to TAM-treated *Klf5*^ΔIND^ mice (Figure 10, E–G). In addition, antibiotic cocktail treatment in TAM-treated *Klf5*^ΔIND^ mice exhibited negligible accumulation of p-STAT3 in the epithelial cells compared with mice given water alone (Figure 10H). Collectively, these results showed that colitis resulting from the absence of epithelial KLF5 was rescued by antibiotic treatment, suggesting that intestinal microbiota played a vital role in mediating the colitis phenotype in these mice.

## Discussion

We previously established a role of KLF5 in maintaining the integrity of the intestinal epithelial barrier function (24, 34). In this study, we delineated the functional and pathological consequences of *Klf5* deletion from the intestinal epithelium — specifically, the changes in immune profiles, inflammatory environment, and microbiota in the colon of mutant mice. The substantial changes in the colon of TAM-treated *Klf5*^ΔIND^ mice, such as crypt distortion and leukocyte infiltration, are highly reminiscent of those observed in human colitis (35). Phenotypically, *Klf5* deletion in the mouse model also recapitulated some clinical findings typical of human diseases, such as blood in the stool and weight loss. As shown from patient specimens, *KLF5* expression exhibited a dose-dependent relationship with disease severity in some patients with IBD (Figure 1).

Our study showed that TAM-treated *Klf5*^ΔIND^ female mice were more susceptible to the development of colitis with higher mortality than male mice. Previous studies have also observed sex-based differences in promoter-driven CreER^T2^, as in the AdipoqCre-ER^T2^ × tdTomato (TDTO) mouse model developed by Lindhorst et al. (36). In their model, administration of TAM results in the activation of TDTO in adipocyte cells, and similar to the current *Klf5*^ΔIND^ mouse model, Lindhorst et al. used a 5-day TAM treatment course. Importantly, they observed increased numbers of adipocytes expressing TDTO in female mice compared with male mice upon TAM treatment (36).

Colonoid cultures from *Klf5*^ΔIND^ mice in our study have shown that *Klf5* is essential in maintaining crypt renewal and expansion. When *Klf5* was deleted, we observed a substantial reduction in the percentage of colonoids that were budding. We attribute this reduced colonoid maintenance to *Klf5*’s role in regulating intestinal stem cell renewal. It was previously documented that *Klf5* deletion reduces intestinal stem cell signature genes, *Lgr5*, *Olfm4*, *Ascl2*, and *Smoc2* (27).

TAM-treated *Klf5*^ΔIND^ mice exhibited a Th17 immune response accompanied by increased production of IL-17A, IL-22, and IL-6 (Figures 3 and 4). This is also similar to the strong correlation between the Th17 response and a subset of patients with IBD (37). Newer therapies in IBD target essential inflammatory cytokines for Th17 cell signaling and maturation; for instance, tocilizumab is a mAb directed against IL-6 (38). IL-6 is an important inflammatory marker that helps the development of Th17 cells (39). Other therapies have also been developed to target hallmark effector cytokines of Th17 to reduce the colonic tissue’s inflammatory response. We have demonstrated the importance of Th17’s effector cytokines in the pathogenesis of colitis by the administration of neutralizing IL-17A to the mutant mice, which resulted in improved intestinal morphology and clinical scores. However, clinical trials with αIL-17A agents in patients with IBD paradoxically induced fulminant IBD (14). We hypothesize the difference in response is due to the *Klf5*^ΔIND^ mouse model representing acute colitis, whereas the patients receiving αIL-17A treatment were experiencing chronic colitis. The discrepancy in response to blocking IL-17A has also been explained by Park et al., who have shown that *Il-17a^–/–^* T cell transfer and αIL-17A treatment can attenuate acute colitis and not chronic colitis (40). Other groups have begun to investigate blocking IL-22 as a possible next target for IBD treatment, since IL-22 can have a pathogenic effect by hyperstimulating Th17 maturation (41). We anticipate our mouse model to be a potentially powerful tool for developing additional therapeutics for IBD, given clinical research trends.

Depending on the context, IL-22 can be proinflammatory or antiinflammatory (20). Mihi et al. demonstrated that IL-22 is essential in promoting intestinal epithelial cell regeneration for necrotizing enterocolitis in neonatal mice (42). They developed an *Il22ra1*-KO model to investigate the effects of perturbed IL-22 signaling. These mice did not have spontaneous colitis or weight loss resulting from the intact epithelial barrier. Interestingly, induction of necrotizing enterocolitis did not significantly increase inflammatory markers between WT and *Il22ra1*-KO mice, but Mihi et al. explained that neonates cannot mount a strong IL-22 response (42). Furthermore, the addition of rIL-22 rescued the phenotype in WT and not *Il22ra1*-KO mice. These results potentially explain the differences in phenotypes between the *Klf5*^ΔIND^ and *Il22ra1*-KO models.

In the intestinal tract, IL-22 produced by Th17 cells can elicit regenerative properties in intestinal epithelial cells, such as the production of antimicrobial peptides and mucin (41). Our study showed that downstream IL-22 signaling in the intestinal epithelial cells is perturbed in the absence of KLF5. Mechanistically, KLF5 is required for the proper nuclear localization of p-STAT3 elicited by IL-22, and this is in part explained by the physical interaction between KLF5 and p-STAT3. Thus, the absence of KLF5 effectively abolishes STAT3 transcription factor functions (20), which leads to the absence of epithelial cell regeneration and antimicrobial peptide production when *Klf5* is deleted. We also demonstrated that *Klf5* reduction or deletion resulted in reduced EdU staining both in vitro and in vivo, another important outcome of p-STAT3.

Colonoid cultures have confirmed the reduction in both proliferation and crypt structure maintenance regardless of rIL-22 presence when KLF5 is deleted. Previous studies have shown conflicting results when rIL-22 is added to enteroid cultures; some have reported rIL-22 being detrimental to enteroid viability (43), although many have suggested otherwise (44). Patnaude et al. have demonstrated proliferation and viability in human colonoid cultures. They showed that rIL-22 can promote epithelial regeneration and innate defense and mucus production (44). Our results are in agreement with those of Patnaude et al., as we observed an increase in proliferation and viability in colonoids with intact, but not deleted, KLF5 after IL-22 treatment.

Downstream targets of IL-22–induced p-STAT3 signaling did not significantly increase in *Klf5*-deleted mice, including S100A8/9, β-defensin 2, and SOCS3. Furthermore, we also demonstrated that aberrant p-STAT3 nuclear localization was not specific to IL-22–induced signaling. IL-6 stimulation, which signals through gp130 and IL-6Rα, also led to aberrant nuclear localization of p-STAT3 in the absence of KLF5. Tetreault et al. had previously described that an increase in *Klf5* expression helped protect DSS-treated mice from colitis by increasing phosphorylation of STAT3 via IL-22 (45). Given the role of KLF5 in regulating critical epithelial cell functions, such as proliferation and regeneration (25), it is not surprising that mice lacking KLF5 failed to develop a regenerative response despite the presence of IL-22.

We have observed several parallel features between our *Klf5*^ΔIND^ mice and patients with UC. First, we observed a dose-dependent response between KLF5 expression and the severity of colitis. Second, our *Klf5*^ΔIND^ mouse colons had similar immunological profiles as the colons of patients with UC. Last, we have shown aberrant localization of p-STAT3 in the intestinal epithelium for both our *Klf5*^ΔIND^ mouse colons and the biopsies of patients with UC. These similarities render the *Klf5*^ΔIND^ mouse model potentially beneficial to the study of human diseases.

The intestinal T cells and the microbiota influence one another to maintain homeostasis. During homeostasis, the microbiota modulates intestinal immunological response with metabolites to promote antiinflammatory responses (46). However, dysbiosis of the intestinal epithelium allows microbe infiltration into the intestinal epithelium, which triggers a proinflammatory environment hostile to commensal bacteria (47). Here, we hypothesized that changes in the microbiome result from inflammation. Such changes were reflected in specific microbial populations in the colon upon *Klf5* deletion. For instance, the population abundance of *Firmicutes*, *Clostridia*, SFB, and BAT were significantly different in TAM-treated *Klf5*^ΔIND^ mice versus control mice. Decreased abundance of *Firmicutes* has been shown to be associated with increased risk for postoperative recurrence of CD (48).

Similarly, 16s rRNA-Seq results of patients with IBD have demonstrated decreased *Clostridia* abundance (49). Furthermore, increases in SFB and BAT have been correlated with patients with UC and CD compared with those in healthy individuals. Both SFB and BAT are pathobionts for Th17 cells. SFB can induce Th17 response by adhering to epithelial cells to activate SAA1/2, and BAT can produce *B*. *fragilis* toxin that can alter tight junctions and increase DC IL-23 activity. Notably, the increased counts of SFB and BAT in the colon of TAM-treated *Klf5*^ΔIND^ mice are reminiscent of that in human diseases too.

**Figure 11:**
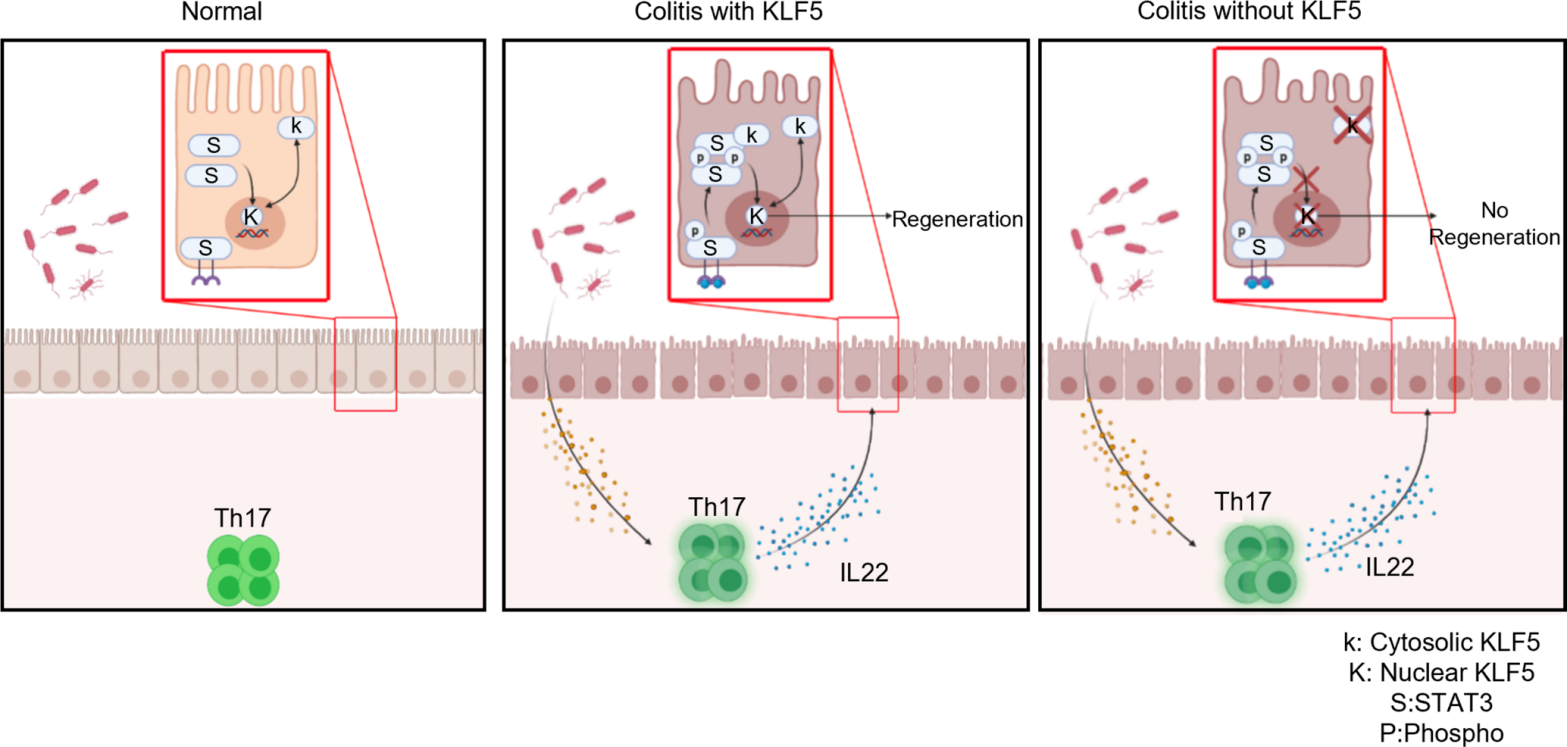
Model for a role of epithelial KLF5 in guarding against intestinal inflammation. Left shows the homeostatic state of the colon in which KLF5 regulates proliferation of crypt epithelial cells (27). Nuclear-cytoplasmic shuttling of KLF5 has previously been demonstrated (56). Middle panel shows colitis with intact KLF5, which promotes IL-22–mediated epithelial regeneration by assisting in the nuclear localization of p-STAT3. Right panel shows colitis in the absence of KLF5 due to the lack of p-STAT3 nuclear localization in response to IL-22.

Deletion of *Klf5* from the intestinal epithelium compromises barrier integrity (24). As a result, the microbiota can infiltrate the intestinal epithelial barrier to induce a Th17 response, which includes IL-22 secretion. We have shown here that microbiota plays an essential role in the pathogenesis of colitis by rescuing the colitic phenotype with antibiotics. During colitis with intact KLF5, IL-22 helps signal regeneration in the intestinal epithelium through p-STAT3 signaling. However, we have demonstrated that regeneration cannot occur independently of KLF5. Based on these findings, we conclude that KLF5 assists in the nuclear localization of p-STAT3 and subsequent downstream signaling for epithelial regeneration. Collectively, these observations support a model where deletion of *Klf5* from the intestinal epithelium results in a persistent colitic phenotype as a consequence of the inability of KLF5 (due to its absence) to regulate p-STAT3 localization in the presence of IL-22–producing Th17 cells required for regeneration (Figure 11).

In summary, deletion of *Klf5* in the intestinal epithelium results in an inflammatory response in mice and an inability to resolve the epithelial injury. The combined insults result in a severe colitic phenotype that impedes survivability. We have also elucidated the mechanism by which KLF5 guards against the development of colitis. These findings strongly support the *Klf5*^ΔIND^ mice as a potentially powerful tool in modeling for human colitis and developing therapeutics for IBD.

## Methods

### Experimental model and animal details

#### Mice.

All animal studies were performed following the protocols approved by the Stony Brook University IACUC. Female and male 8- to 12-week-old *Villin-CreER^T2^* (*Klf5^Ctrl^*) and *Villin-CreER^T2^ Klf5^fl/fl^* (*Klf5*^ΔIND^) C57BL/6 mice were previously described and bred in-house (24).

#### Mouse treatments.

Cre^ERT2^-recombinase activity was induced by the administration of 100 μL of 1 mg TAM for 5 consecutive days. TAM was dissolved in CO; thus, CO was used as a control treatment (24). Cocktail antibiotic treatment with metronidazole (100 mg/kg BW) (50), ciprofloxacin (50 mg/kg BW) (51), and vancomycin (50 mg/kg BW) (50) was also performed concurrently with CO or TAM by oral gavage.

#### IL-17A neutralization.

Neutralization of IL-17A with αIL-17A antibody (BioXCell *InVivo*MAb anti-mouse IL-17A, BE0173) was performed in 8-week-old *Klf5*^ΔIND^ female mice. Mice were treated with CO/TAM injections concurrently with IgG1 (BioXCell *InVivo*MAb mouse IgG1 control, BE0083) or αIL-17A at 100 μg/mL for 5 consecutive injections. One day after the last injection, colons were collected for histology and downstream analysis.

### Lamina propria and intestinal epithelial cell isolation and FACS

Isolation of leukocytes was performed 24 hours after the fifth dose of CO or TAM. The protocol was performed as previously published (52). Staining was performed with primary antibodies conjugated with an array of fluorochromes. Intestinal epithelial cells were isolated with EDTA and stained for EpCAM, p-STAT3, and STAT3. A list of all FACS antibodies is in Supplemental Table 1. The acquisition was performed on a BD Biosciences LSRFortessa at Stony Brook University Flow Cytometry Research Core Facility. Analysis was performed with FlowJo.

### Effector cytokine FACS

After isolation of leukocytes from the lamina propria, cells were stimulated with the polyclonal stimulus PMA and ionomycin in the presence of brefeldin A for 4 hours (53). Cells were then stained for cell surface markers. Fixation and permeabilization were performed with eBioscience Transcription Factor Staining Buffer Set (catalog 00-5523-00) followed by intracellular cytokine staining.

### Cell lines

C2BBE cell lines were purchased from ATCC and cultured according to their instruction. C2BBE^ΔIND^ was generated as previously described (24).

### Cell line treatment

C2BBE^Ctrl^ and C2BBE^ΔIND^ were all cultured to 50% confluence and then treated with DMSO or DOX (5 μg/mL) for 3 consecutive days. On the final treatment day, medium containing DMSO or DOX was removed, and fresh medium with PBS or rIL-22 (100 ng/mL) was added for 1 hour. Treatments with rIL-6 (100 ng/mL) and with DMSO or DOX were given at the same time. Cells were then collected for protein extraction and IF imaging.

### Cell cycling analysis

After treatment with DMSO/DOX and PBS/rIL-22, cells were trypsinized and fixed in 70% ethanol. They were then stained with propidium iodide, and cells were acquired on BD Biosciences FACSCalibur LSR and analyzed with ModFit LT.

### Western blot analysis

Total protein was extracted from tissue and cell lines with RIPA buffer. Tissue was processed with 300 μL of RIPA buffer for 5 mg of tissue. Cell lines were treated with 1 mL of RIPA buffer for an 80% confluent 10 cm^2^ plate. Laemmli buffer was used to denature and load Western blot gels. Total protein concentration was performed with BCA analysis. A list of antibodies is shown in Supplemental Table 2.

### ELISA

Total protein was extracted from tissue and cell lines with RIPA buffer (Thermo Fisher Scientific). Tissue was processed with 300 μL of RIPA buffer for 5 mg of tissue. Cell lines were treated with 1 mL of RIPA buffer for an 80% confluent 10 cm^2^ plate. Prior to incubating samples on ELISA plates, samples were normalized with BCA analysis of total protein concentration. The protocol of ELISA was provided by R&D Systems.

### RNA extraction, RT-qPCR, and sequencing

RNA extraction for colon tissue was extracted with TRIzol reagent (Thermo Fisher Scientific). The RT-qPCR assay was performed with the TaqMan Gene Expression Master Mix and QuantStudio 3 qPCR machine (both Thermo Fisher Scientific). The following gene expression assays were used: Mm00446190_m1 (*Il-6*), Mm00439618_m1 (*Il-17a*), Mm01226722_g1(*Il-22*), Mm01168134_m1, (*Ifng*), Mm01324470_m1 (*Lcn2*), Mm00487724_m1 (*Mmp7*), Mm00440616_g1, (*Reg3b*), Mm00441127_m1 (*Reg3g*), Mm00441242_m1 (*Ccl2*), and Mm02619580_g1 (*Actb*). The New York Genome Center performed cDNA library construction and high-throughput sequencing. Analysis for sequencing was performed with the HISAT2 pipeline. The initial alignment was performed with HISAT2, transcript counts were quantified with FeatureCounts, and differential gene analysis was performed with DESeq2. Downstream analysis was performed with GSEA for pathway analysis and HOMER for motif scanning (33).

### Histology

Colon tissue from mice was FFPE; 5 μm sections were used for H&E staining (54). All micrographs were captured using a Nikon Eclipse 90i microscope. Human tissue microarray, CO245a, was purchased from US Biomax, and moderate and severe colitis biopsies were obtained from Stony Brook University Biobank.

### IHC and IF

IHC and IF were performed as previously described (24, 34). A list of antibodies is shown in Supplemental Table 2.

### IP

C2BBE and HEK293T HA-hKLF5 cells (ATCC) were treated with rIL-22 (100 ng/mL) for 1 hour. We used Active Motif Nuclear Complex Co-IP kit, and the isolation protocol was performed as instructed in the kit. Antibody pulldown for C2BBE was performed with rabbit anti-KLF5 antibody. Immunoprecipitation of HEK293T HA-hKLF5 was performed with mouse anti-HA antibody. Western blot was performed to visualize KLF5 pulldown and p-STAT3.

### Microbiome analysis

Fecal matter was collected from CO-treated or TAM-treated 8-week-old *Klf5*^ΔIND^ female mice after 5 days of injection. Samples were collected from the ileum and sent to Weill Cornell University Microbiome Core Lab. Analysis was performed in Nephele NIAID pipeline using bioBakery suite. Quantifications of genus and order were obtained from MetaPhlan2 and analyzed with LefSE for LDA score.

### Cell isolation for enteroid culture

Colons were extracted from 8-week-old *Klf5*^ΔIND^ female mice. Crypt cells were isolated as previously described (27, 55). All crypts were embedded in Matrigel (Corning). Colonoid culture media was prepared with L-WRN cells (ATCC) as previously described, and TGF-β inhibitor A83-01 (500 nM; Tocris Biosciences) and antibiotic cocktail Primocin (100 μg/mL; Thermo Fisher Scientific) were supplemented. GSK3β inhibitor CHIR99021 (10 μM; Tocris) and ROCK inhibitor Y-27632 (10 μM; MilliporeSigma) were added only on the first 2 days of culture. After the first 2 days, CHIR99021 and Y-27632 were removed, and PBS or 4-OHT (10 μM) was added. For rIL-22 cultures, PBS/4-OHT was added with PBS/rIL-22 (100 ng/mL).

### Statistics

Statistical analysis was performed on GraphPad Prism Version 9.0.0 for Mac. *P* values of less than 0.05 were considered significant. Statistical tests are reported in the figure legends.

### Reporting summary

#### Data and materials availability.

The accession number for RNA-Seq and 16s rRNA-Seq data deposited in NCBI’s GEO is GSE179310. Further information and requests for resources and reagents should be directed to and will be fulfilled by the corresponding author, VWY.

#### Study approval.

All animal experiments were performed in accordance with recommendations in the NIH *Guide for the Care and Use of Laboratory Animals* (National Academic Press, 2011), as approved by the IACUC (protocol 354918) at Stony Brook University in New York. Patient consents were waived as surgical tissue samples from the biobank were deidentified specimens.

## author contributions

JS, THC, YL, JK, ARDS, and SK performed the experiments; JS, THC, YL, JK, ARDS, and SK analyzed the data; SYZ assisted in histological evaluation of tissue; JS, THC, YL, JK, ARDS, SK, BSS, ABB, and VWY provided conceptual input; JS, ABB, and VWY wrote the paper; ABB and VWY conceived and supervised the study.

## Supplementary Material

Supplemental data

## Version 1 04/08/2022

Electronic publication
