# Supplementary material for: KLF5 protects the intestinal epithelium against Th17 immune response in a murine colitis model
Source: JCI Insight. 2022 Apr 8;7(7):e153488. doi: 10.1172/jci.insight.153488 (PMC9057631; doi:10.1172/jci.insight.153488)
Supplement: Supplemental data [file jciinsight-7-153488-s064.pdf]

SUPPLEMENTAL FIGURE LEGENDS

Supplemental Figure 1: The effect of *Klf5* deletion on the intestinal phenotypes in male and female mice. (A) Kaplan-Meier curve of CO and TAM treatment for *Klf5*$^{\Delta IND}$ mice with male (n=15) and female (n=15) mice. (B) Quantification of clinical scores of CO- or TAM-treated *Klf5*^{Ctrl} and *Klf5*$^{\Delta IND}$ male and female mice (n=10). Max for the clinical score is 12 and quantified with three categories; stool consistency, weight loss, and fecal blood. (C) Hematoxylin-eosin staining of whole colon tissue from *Klf5*^{Ctrl} and *Klf5*$^{\Delta IND}$ male mice treated with CO or TAM. (D) Quantification of histological score of CO- and TAM-treated *Klf5*^{Ctrl} and *Klf5*$^{\Delta IND}$ male and female mice. Max for the histological score is 11 and quantified with three categories; crypt damage, inflammatory cells in lamina propria, and ulcers (n=10). (E) Hematoxylin-eosin staining of ileal tissue from *Klf5*^{Ctrl} and *Klf5*$^{\Delta IND}$ male and female mice treated with CO or TAM. The red square highlights areas of villus blunting in the small intestine of TAM-treated female *Klf5*$^{\Delta IND}$ mice. Data from graphs represent mean with SEM. **P<0.01; ***P<0.001; One-way ANOVA. Scale Bars on images indicate 70μm.

Supplemental Figure 2: Different recombination efficiencies of *Klf5* deletion between male and female *Klf5*$^{\Delta IND}$ mice. (A) Immunohistochemical staining of KLF5 in whole colon tissues from male *Klf5*^{Ctrl} and *Klf5*$^{\Delta IND}$ mice treated with CO or TAM. (B) RT-qPCR analysis of *Klf5* transcript levels comparing TAM-treated over CO-treated *Klf5*$^{\Delta IND}$ mice in male and female (n=9). (C) DNA gel electrophoresis of PCR products from primers flanking *loxP* sites upstream of *Klf5* exon 2 and *Actb*. Data from graph B represent mean, ***P<0.001; Unpaired T-test. Data from graph D represent mean, *P<0.05; ***P<0.001; One-way ANOVA. Scale bars on images indicate 70μm.

Supplemental Figure 3: Comparison of transcriptional profiles between CO- or TAM-treated *Klf5*^{Ctrl} and *Klf5*$^{\Delta IND}$ mice. (A) RT-qPCR analysis comparing CO- to TAM-treated *Klf5* ^{Ctrl} mice (n=3). (B) RT-qPCR analysis comparing CO-treated *Klf5* ^{Ctrl} mice, and CO-treated *Klf5* $^{\Delta IND}$

mice (n=3). (C) RT-qPCR analysis comparing TAM-treated *Klf5*^{Ctrl} mice and TAM-treated *Klf5*$^{\Delta IND}$ mice (n=3). (D) GSEA pathway analysis positively correlated to CO-treated *Klf5*$^{\Delta IND}$ mice. Data from graphs represent mean with SEM. ***P<0.001; Unpaired T-test.

Supplemental Figure 4: Gating strategy for pan-leukocyte flow analysis. Gating strategy for identification of CD45, neutrophil, B cell, T cell, NK cells, monocytes, macrophages, and dendritic cells.

Supplemental Figure 5: Comparison of additional leukocyte populations between CO- or TAM-treated *Klf5*^{Ctrl} and *Klf5*$^{\Delta IND}$ mice. (A) NK cells, (B) B cells, (C) monocytes, (D) neutrophils, (E) macrophages, (F) cDC1, (G) Th1, (H) Th2, and (I) ILC3 cells (n=4). (J) 40X brightfield image of ileum and colon of TAM-treated *Klf5*$^{\Delta IND}$ mice. Red arrows highlight neutrophil involvement in cryptitis and crypt abscesses. Data from graphs represent mean with SEM. The small intestine image of TAM-treated *Klf5*$^{\Delta IND}$ mice in Supplemental Figure 5J is a zoomed-in image of a section of the ileal sample from TAM-treated female *Klf5*$^{\Delta IND}$ mice in Supplemental Figure 1E. One-way ANOVA. Scale Bar represents 70 μm.

Supplemental Figure 6: Comparison of Th17-inducing factors between CO- or TAM-treated *Klf5*$^{\Delta IND}$ mice. *Klf5*$^{\Delta IND}$ mice were treated with CO or TAM for five days and RT-qPCR analysis performed on whole colon tissues for (A) IL-1β , (B) IL-23, and (C) SAA. Cytokine array analyses were performed at days 1 and 5 of CO or TAM treatment for (D) IL-1β , (E) IL-23, and (F) SAA. (G) ELISA of IL-23 concentrations on days 1, 3, 5, and 7 following CO or TAM treatment. Data from graphs A-F represent mean, *P<0.05; **P<0.01; One-way ANOVA test. Data from graph G represent mean, ****P<0.0001; Two-way ANOVA test.

Supplemental Figure 7: Effects of *Klf5* deletion on pSTAT3-related upstream and downstream signaling pathways. (A) Immunohistochemical staining of STAT3 and phosphorylated STAT3 in the colon of CO- or TAM-treated *Klf5*$^{\Delta IND}$ mice. Scale bars on images indicate 70µm. The pSTAT3 images are lower magnifications of the same images shown in Figure 6A. (B) Quantification of percentage of pSTAT3^{+} cells per crypt (n=5). (C) RNA-seq (left graph; n=4) and RT-qPCR (right graph; n=7) analysis of IL-22RA1 transcript levels. (D) RNA-seq (left graph; n=4) and RT-qPCR (right graph; n=6) analysis of IL-22RA2 levels. (E) RNA-seq TPM fold-change for S100A8, S100A9, b-defensin-2, and SOCS3 9n=4). Data from graph B represent mean. ***P<0.001; One-way ANOVA test. Data from graph C-E represent mean by unpaired T-test. Scale bar represents 70µm.

Supplemental Figure 8: Effect of KLF5 deletion on pSTAT3 localization in response to IL-6. IF staining of DAPI and pSTAT3 of C2BBE^{Ctrl} and C2BBE$^{\Delta IND}$ cells treated with DMSO or DOX and PBS or IL-6. Scale bar represents 70µm.

Supplemental Figure 9: Cellular localization of pSTAT3 in human colonic specimens. IHC staining of pSTAT3 in colonic biopsy specimens from normal subject, UC patient, and patient with colon adenocarcinoma. Scale bars represent 70µm.

Supplemental Figure 10: The role of KLF5 in the maintenance and survival of colonoids. (A) Western blots and (B) quantification of KLF5 levels in colonoid cultures from *Klf5*$^{\Delta IND}$ mice treated with PBS or 4-OHT (n=6). (C) Bright field images of colonoid culture from days 1, 3, and 5 of treatment with PBS or 4-OHT. Scale bars on images indicate 70µm. (D) Quantification of percentages of budding colonoids over total number of colonoids on days 0, 1, 3, and 5 (n=8).

Data from graph B represent mean. ****$P<0.001$; Unpaired T-test. Data from graph D represent mean, *$P<0.05$; **$P<0.01$; Two-way ANOVA. Scale bars on images indicate 70μm.

Supplemental Figure 11: Effect of IL-22 on survivability of colonoids derived from *Klf5*$^{\Delta IND}$ mice. (A) Bright field images of *Klf5*$^{\Delta IND}$ colonoid cultures from days 1 and 5 of treatment with PBS or 4OHT. Scale bars represent 70μm. (B) Quantification of percentages of budding colonoids over total number of colonoids on days 1 and 5 (n=10). Data from graph B represent mean, *$P<0.05$; **$P<0.01$; Two-way ANOVA.

Supplemental Figure 12: Comparison of phylogenetic classes in colonic microbiota between CO- and TAM-treated *Klf5*$^{\Delta IND}$ mice. Linear discriminant analysis of 16s rRNA sequencing in the microbiota from the colon of CO- and TAM-treated *Klf5*$^{\Delta IND}$ mice (n=9).

Supplemental Figure 13: Comparison of colonic microbiota abundance in CO- or TAM-treated mice. (A) Phylogenetic tree of LDA analysis comparing TAM-treated *Klf5*^{Ctrl} and *Klf5*$^{\Delta IND}$ mice (n=9). (B) LDA score graph showing significantly different phylogenetic orders between TAM treated *Klf5*^{Ctrl} and *Klf5*$^{\Delta IND}$ mice (n=4). (C) Phylogenetic tree of LDA analysis comparing CO- and TAM-treated *Klf5*^{Ctrl} mice. (D) LDA score graph showing significantly different phylogenetic orders between CO and TAM treated *Klf5*^{Ctrl} mice (n=4). (E) Phylogenetic tree of LDA analysis comparing CO treated *Klf5*^{Ctrl} and *Klf5*$^{\Delta IND}$ mice. (F) LDA score graph showing significantly different phylogenetic orders between CO treated *Klf5*^{Ctrl} and *Klf5*$^{\Delta IND}$ mice (n=9).

Supplemental Figure 14: Effect of *Klf5* deletion on abundance of two Th17 pathobionts. qPCR analysis of 16s rRNA of segmented filamentous bacteria (SFB) (A) and Bacteroides (BAT)

(B) in the colon of CO- or TAM-treated mice (n=6). Data from graph represent mean. *P<0.05; **P<0.01; One-way ANOVA.

Supplemental Figure 15: Effect of antibiotic treatment on the weight of CO- and TAM-treated *Klf5*$^{\Delta IND}$ mice. (A) Raw quantification of weight for each mouse in the experiment (n=3). (B) IHC staining of KLF5 in proximal and distal colons from CO- and TAM-treated *Klf5*$^{\Delta IND}$ mice with water or antibiotic treatment. Scale Bars on images indicate 70μm.

SUPPLEMENTAL TABLE LEGENDS

Supplemental Table 1: List of fluorophore-conjugated antibodies for FACS analysis. and Catalog number, clone, and fluorophores are listed to the right of each antibody marker.

Supplemental Table 2: List of primary antibodies used for IF staining and western blot. The catalog number, clone, and concentrations used for each type of staining protocol.

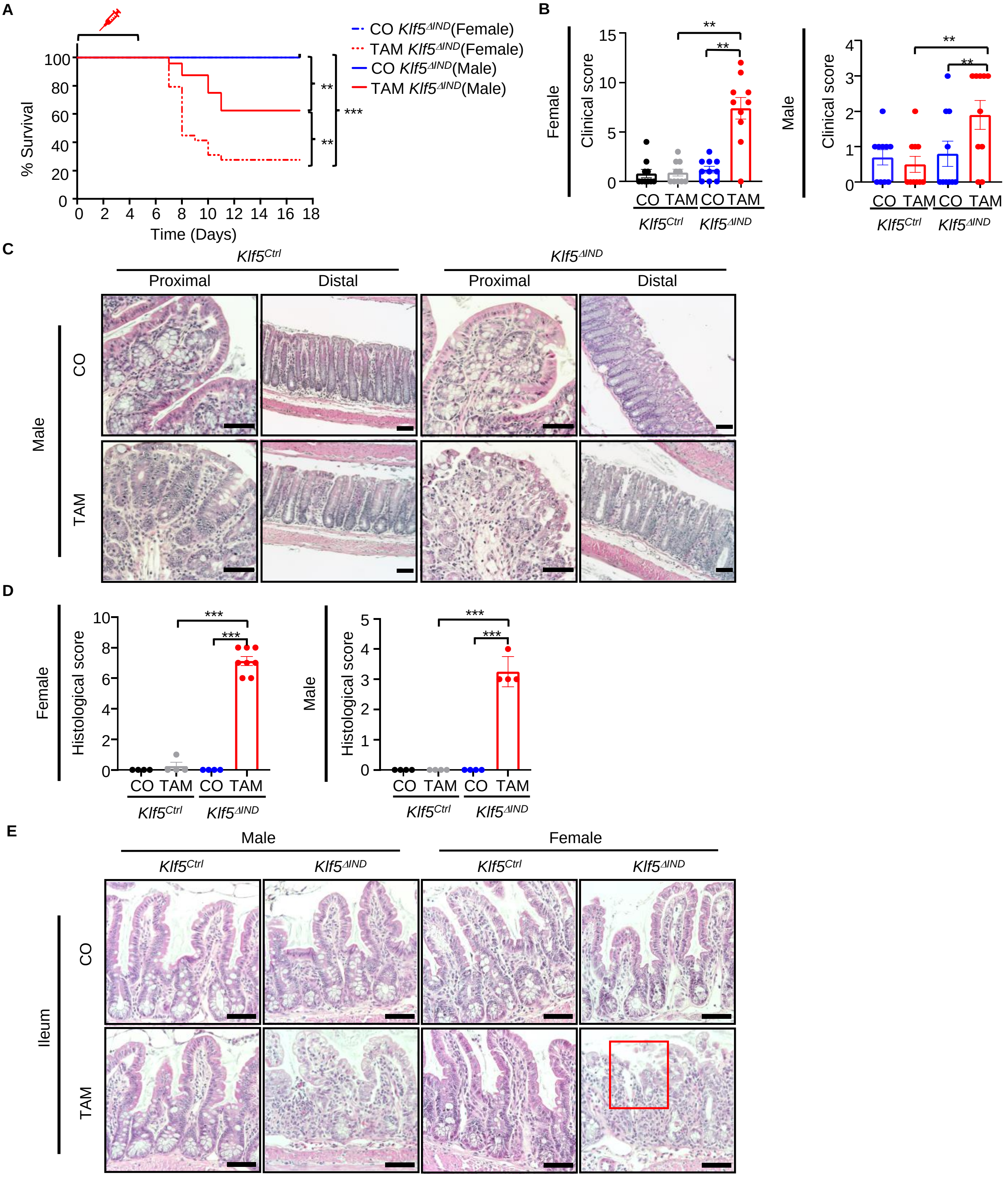

Supplemental Figure 1

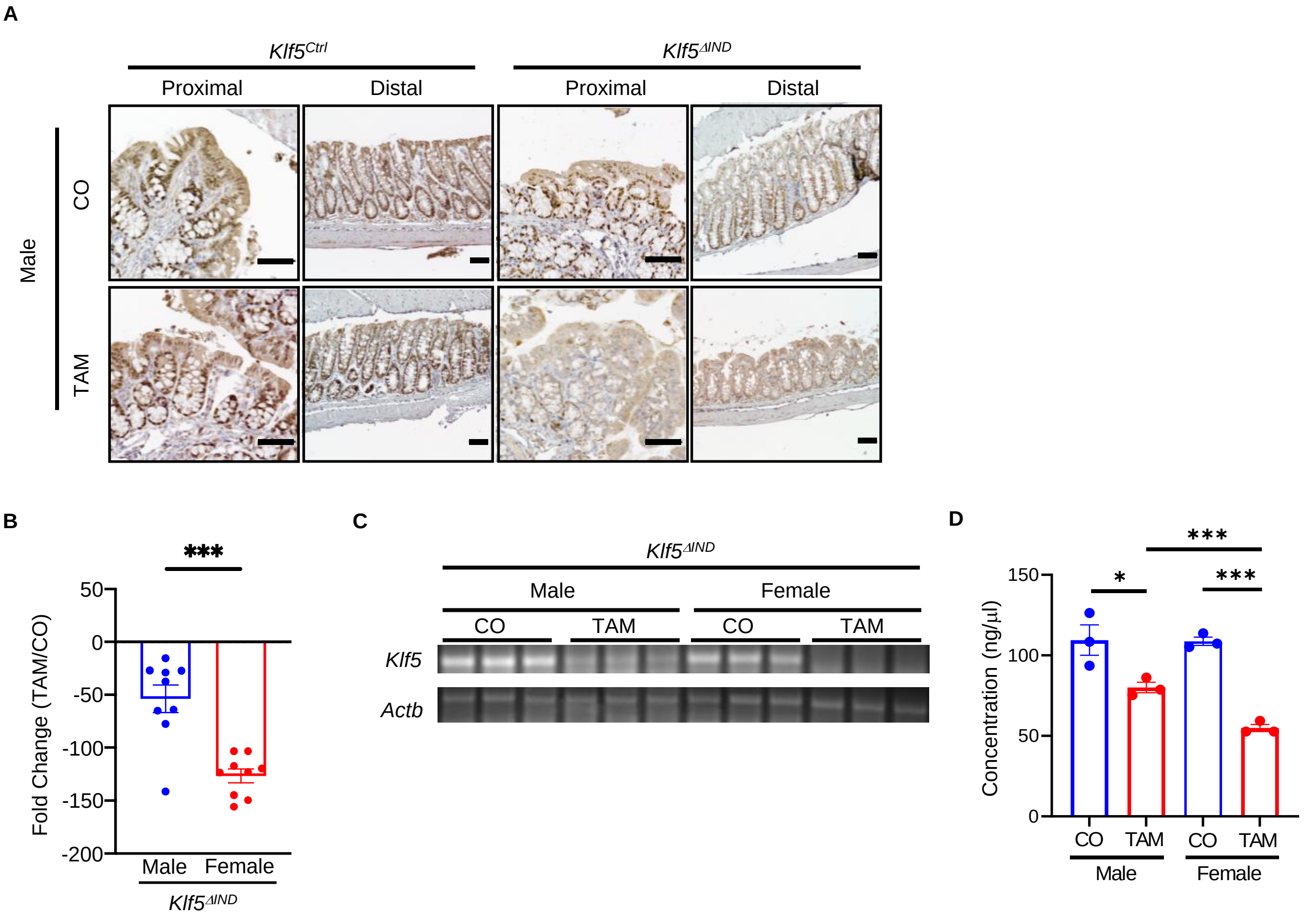

Supplemental Figure 2

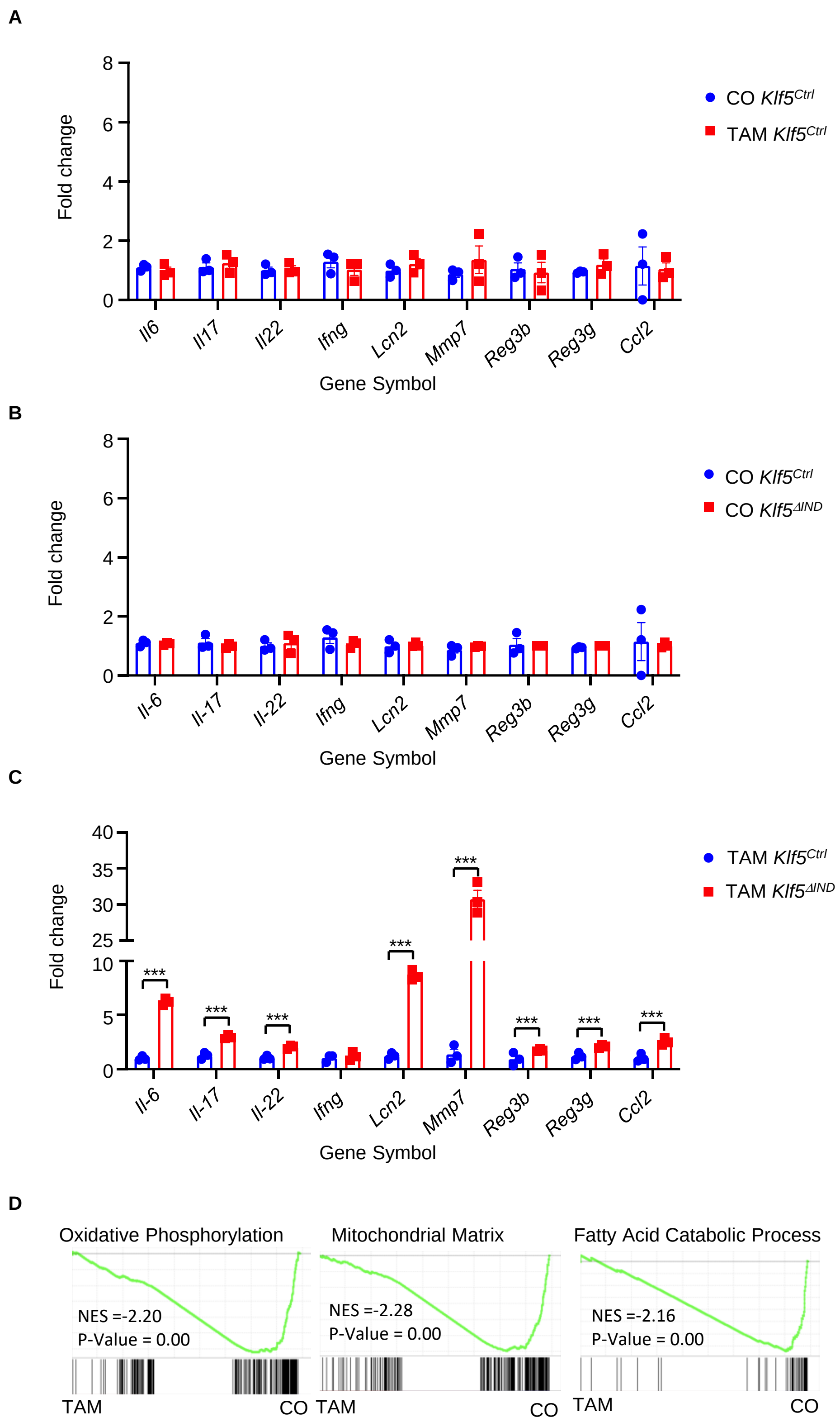

Supplemental Figure 3

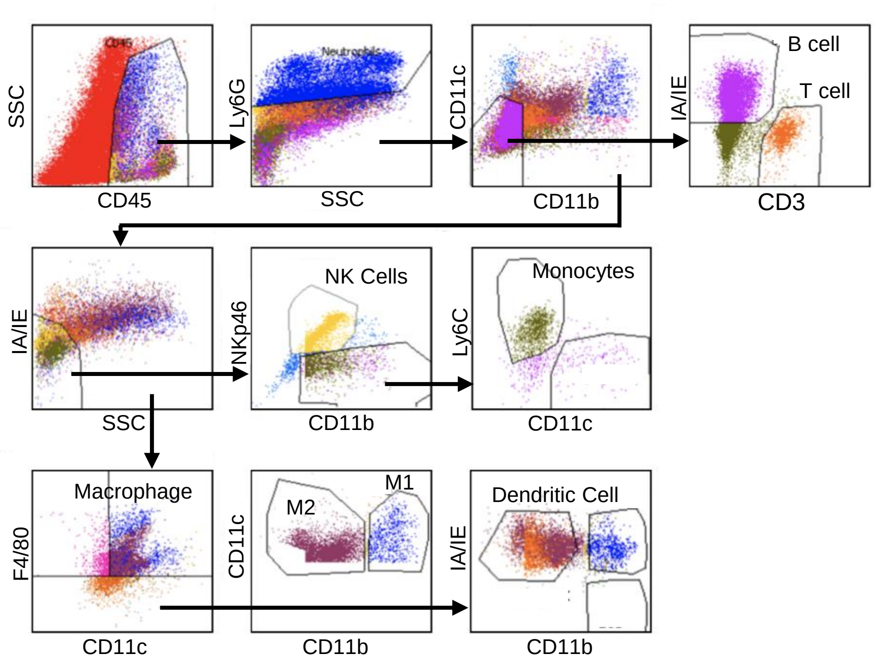

Supplemental Figure 4

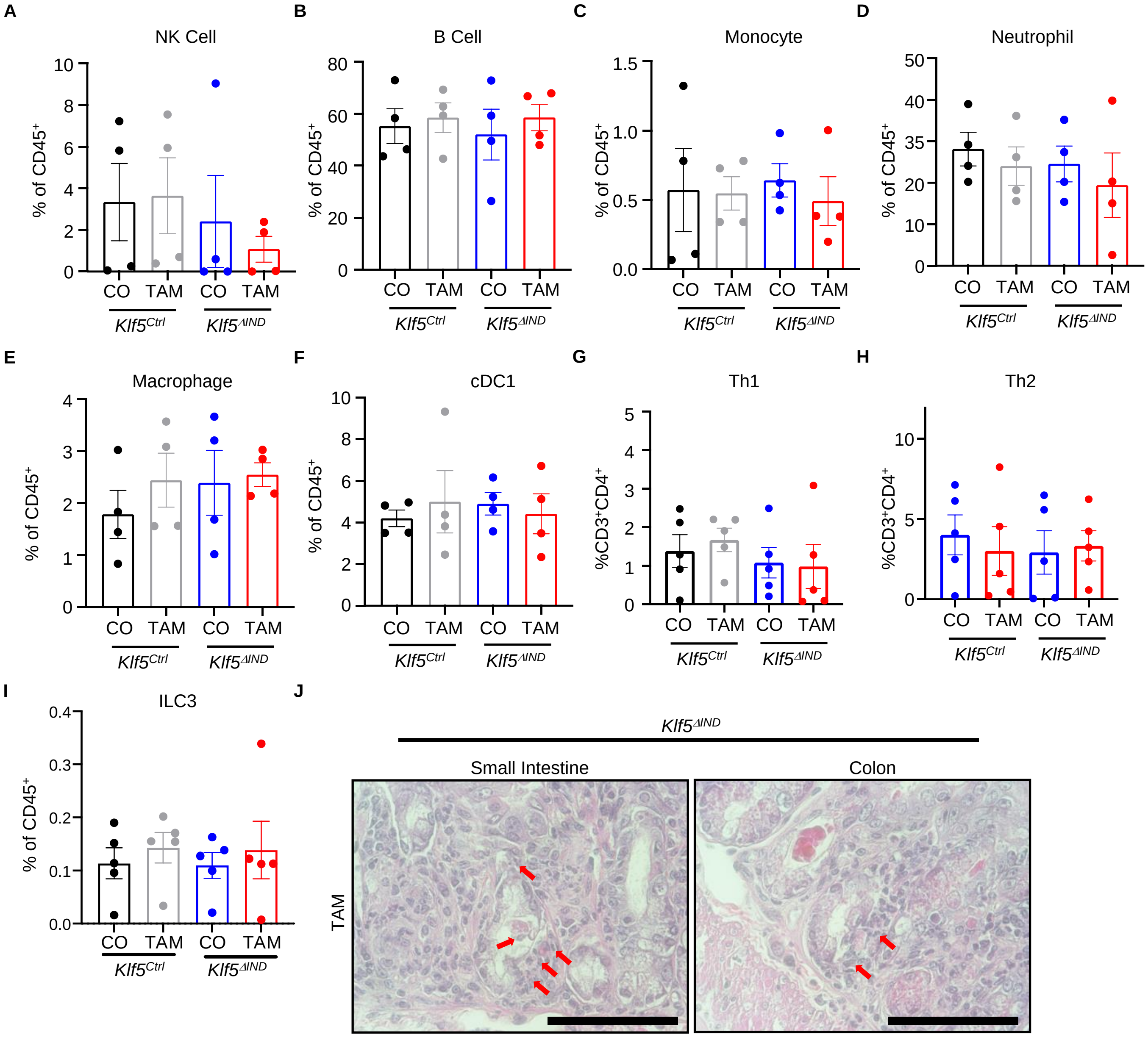

Supplemental Figure 5

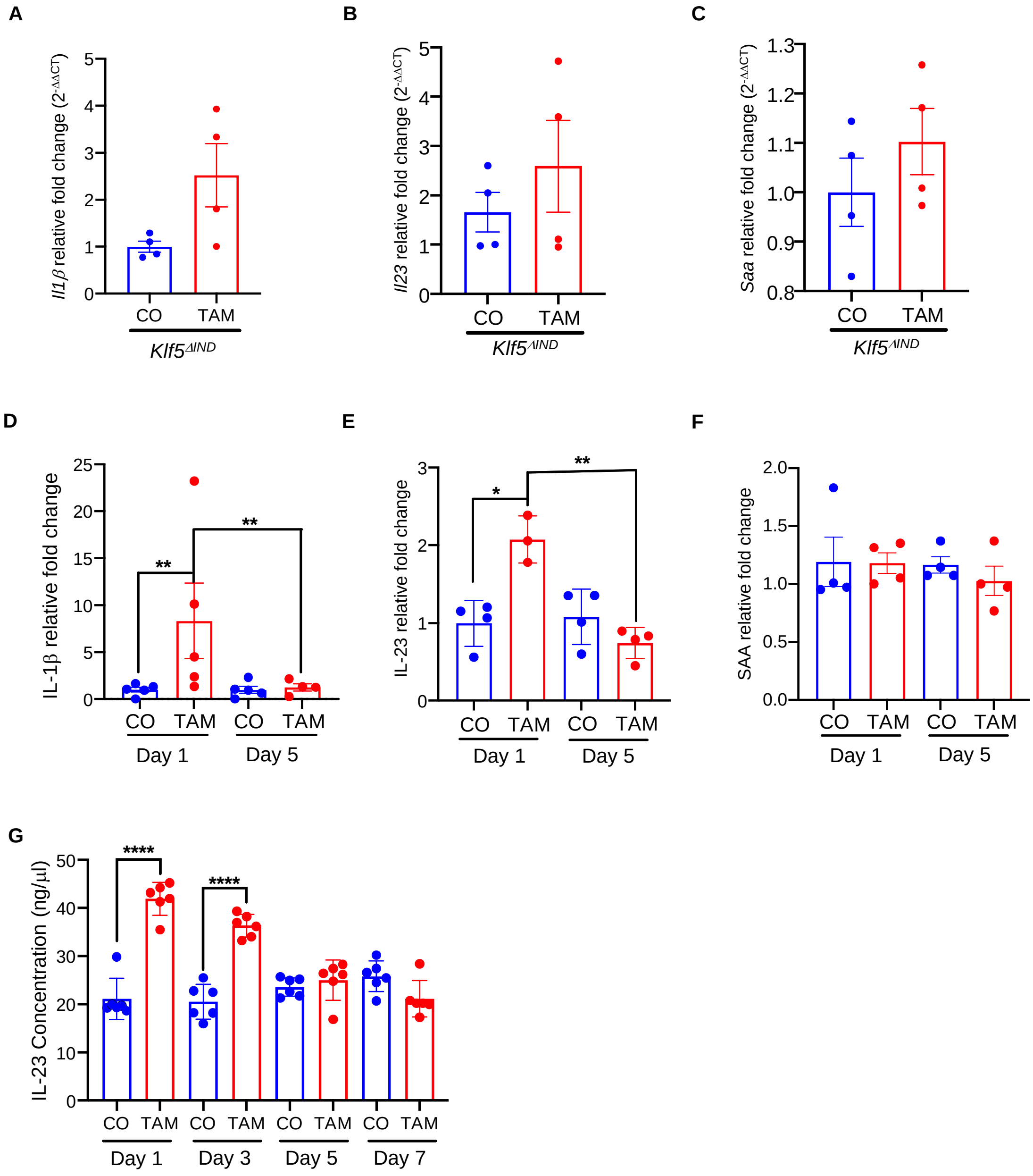

Supplemental Figure 6

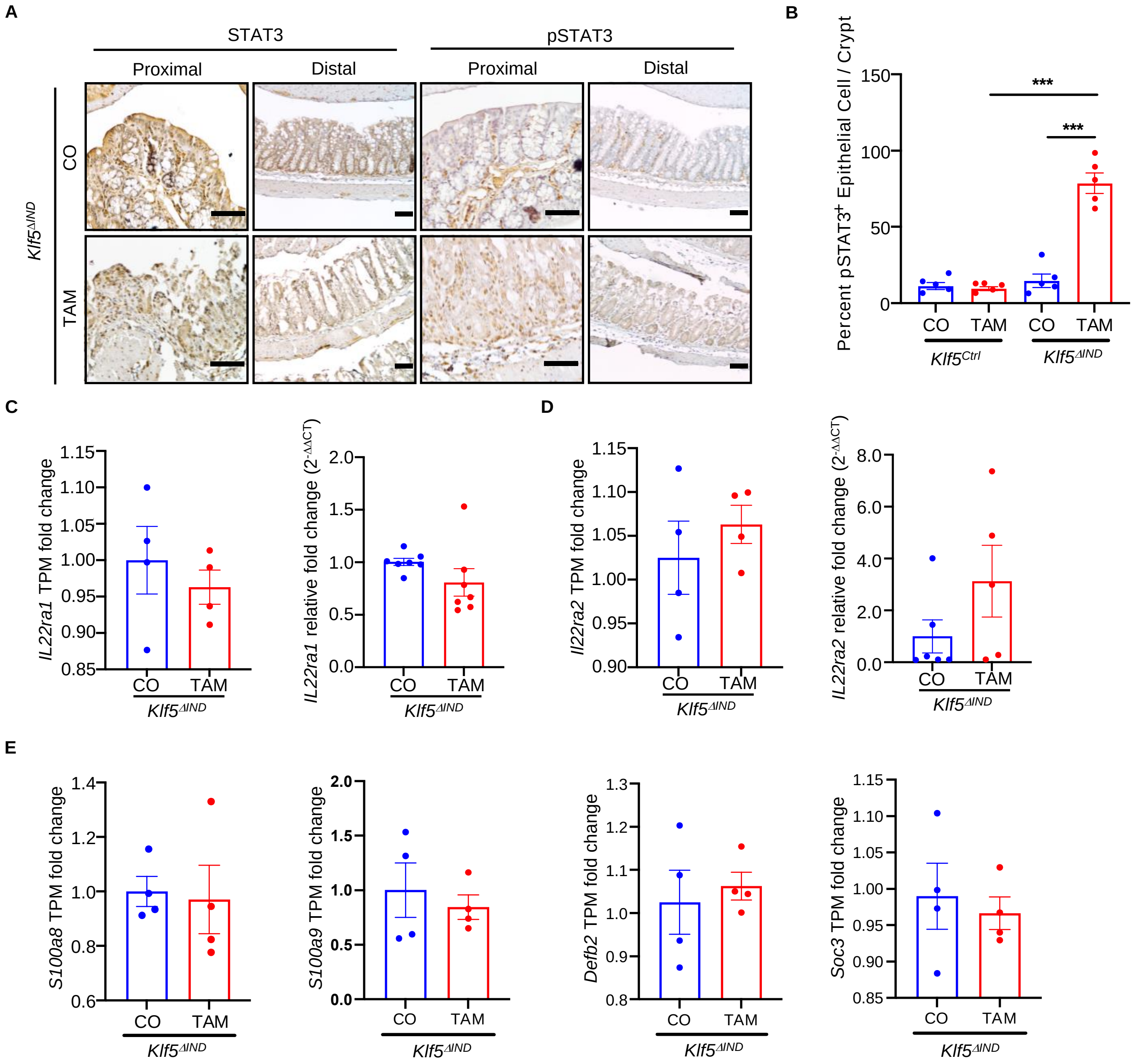

Supplemental Figure 7

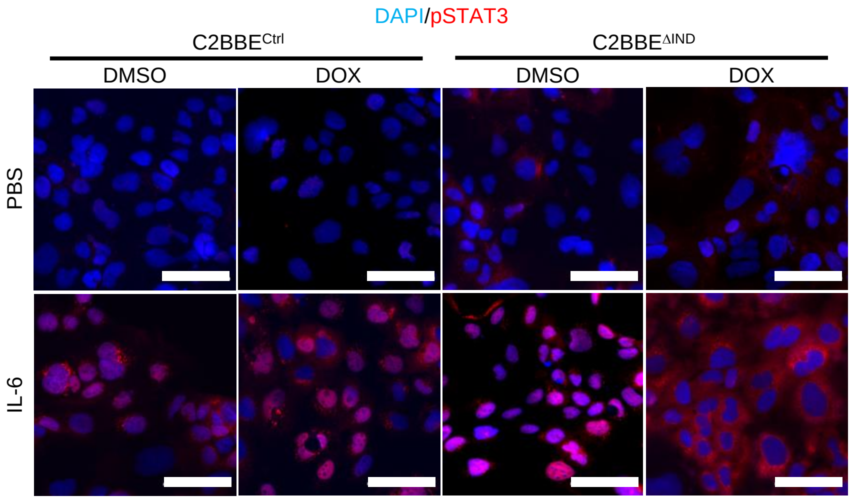

Supplemental Figure 8

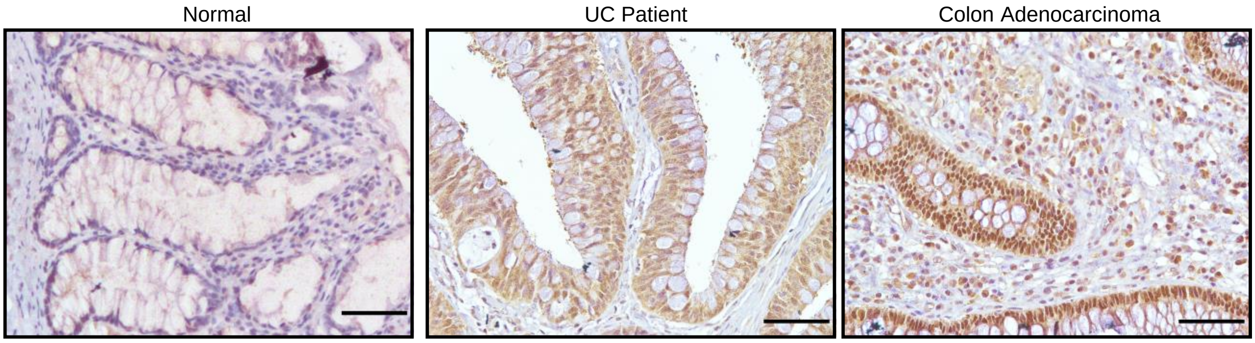

Supplemental Figure 9

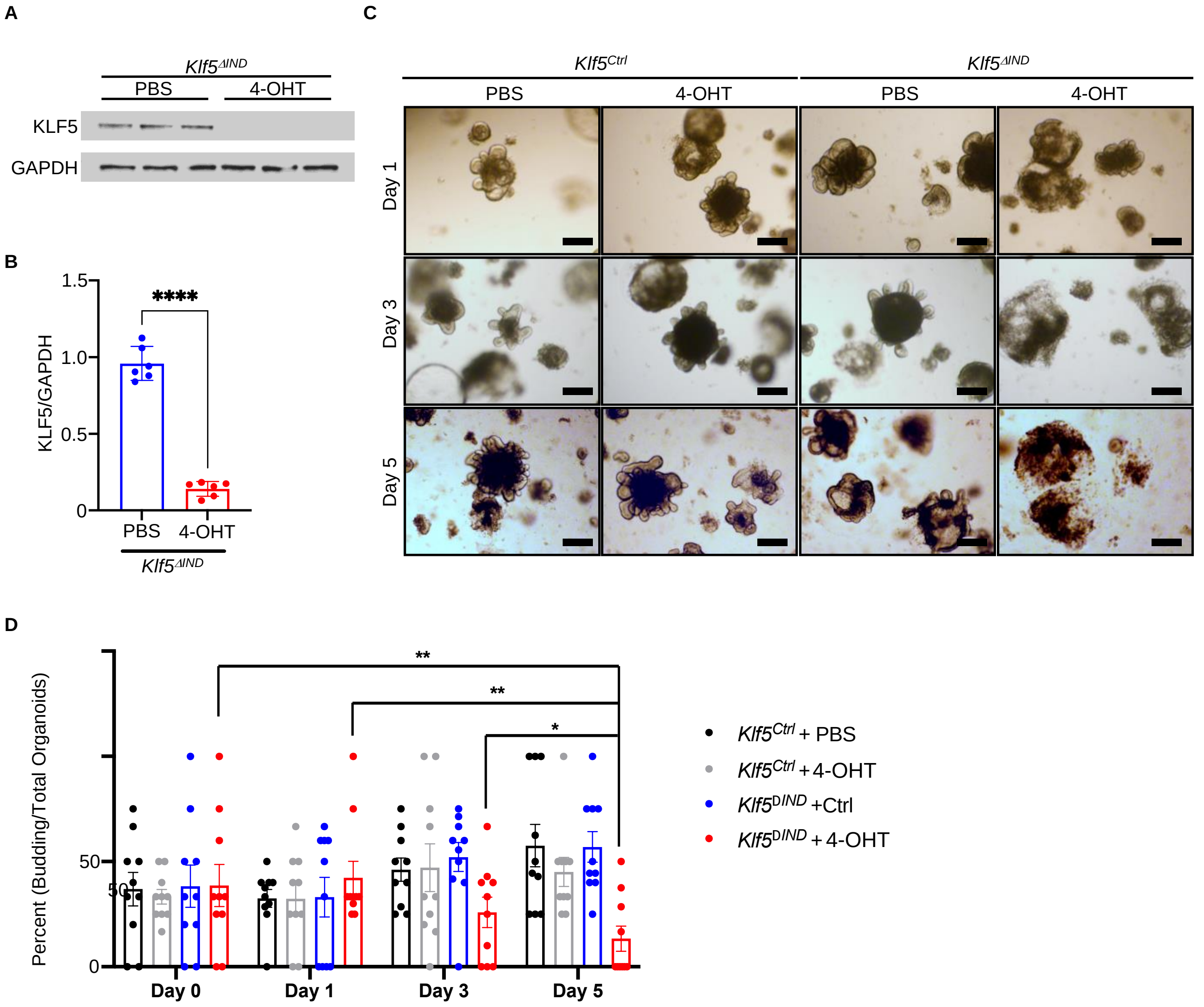

Supplemental Figure 10

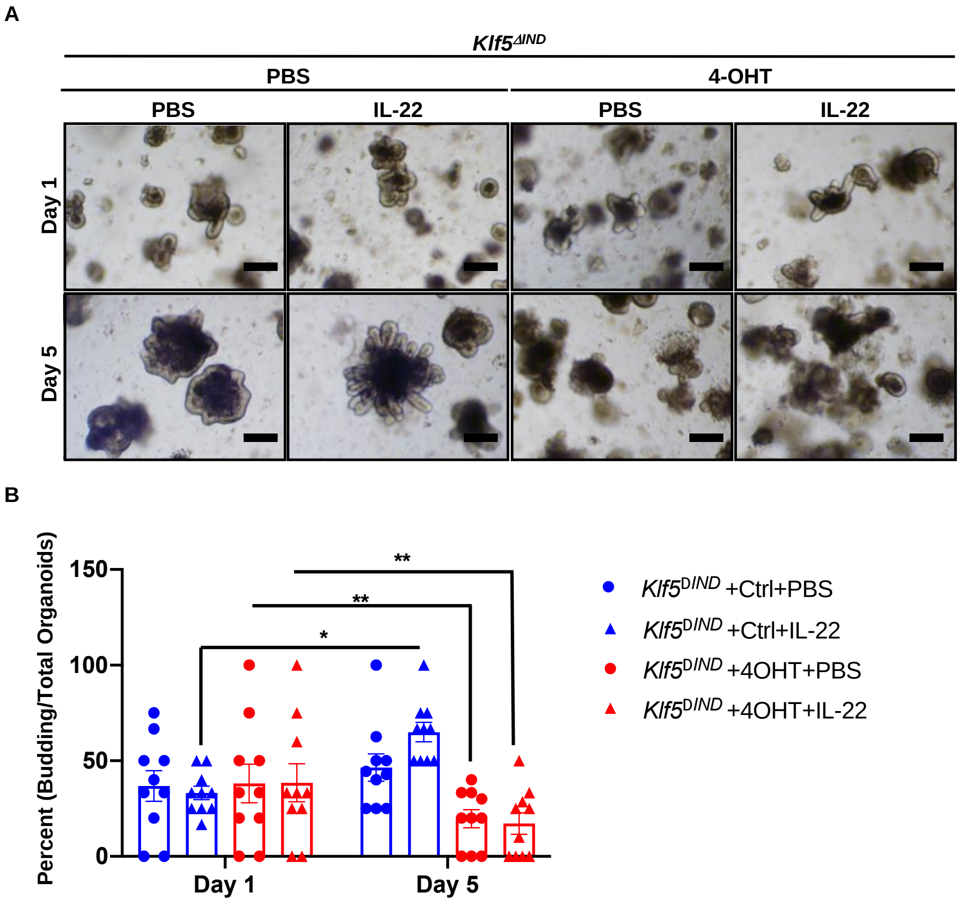
A
Klf5^ΔIND
PBS
4-OHT
PBS
IL-22
PBS
IL-22
Day 1
Day 5
B
Percent (Budding/Total Organoids)
150
100
50
0
Day 1
Day 5
*
**
**
Klf5^DIND +Ctrl+PBS
Klf5^DIND +Ctrl+IL-22
Klf5^DIND +4OHT+PBS
Klf5^DIND +4OHT+IL-22

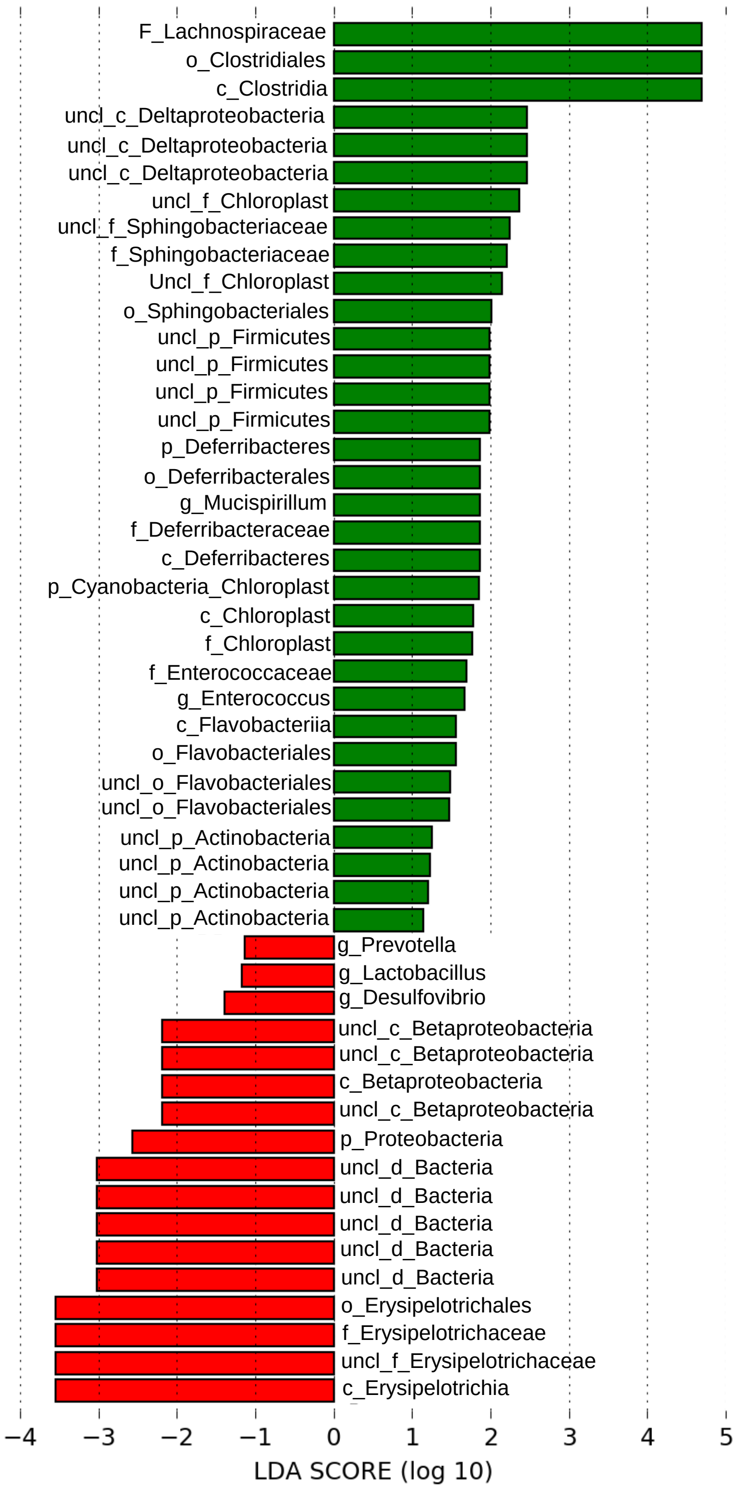

Supplemental Figure 12

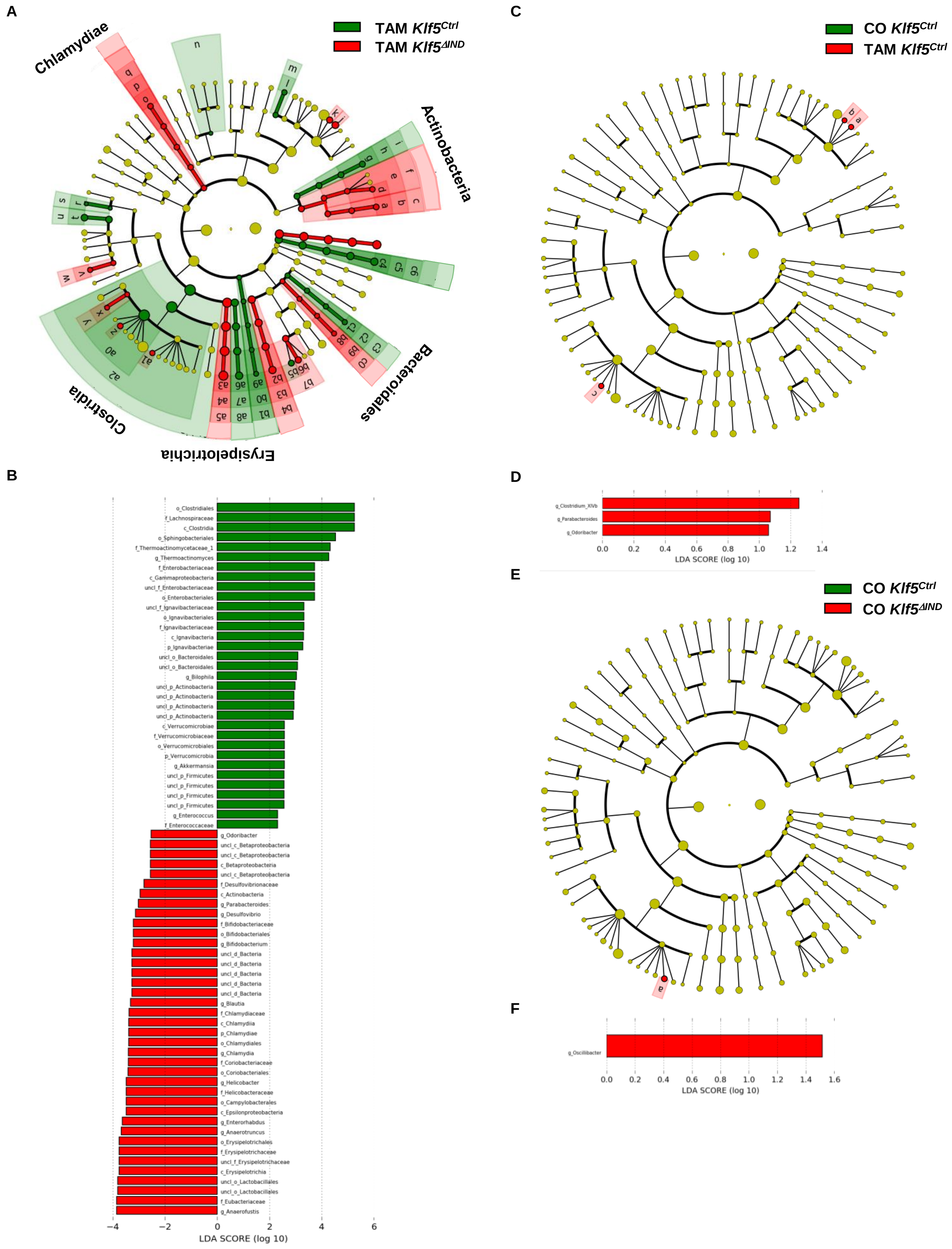

Supplemental Figure 13

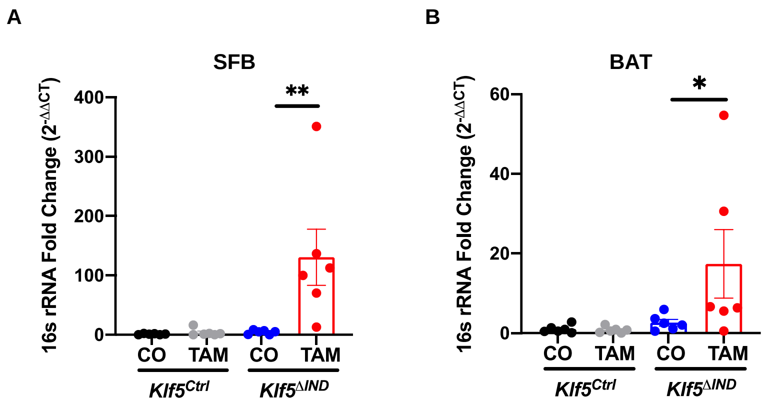

Supplemental Figure 14

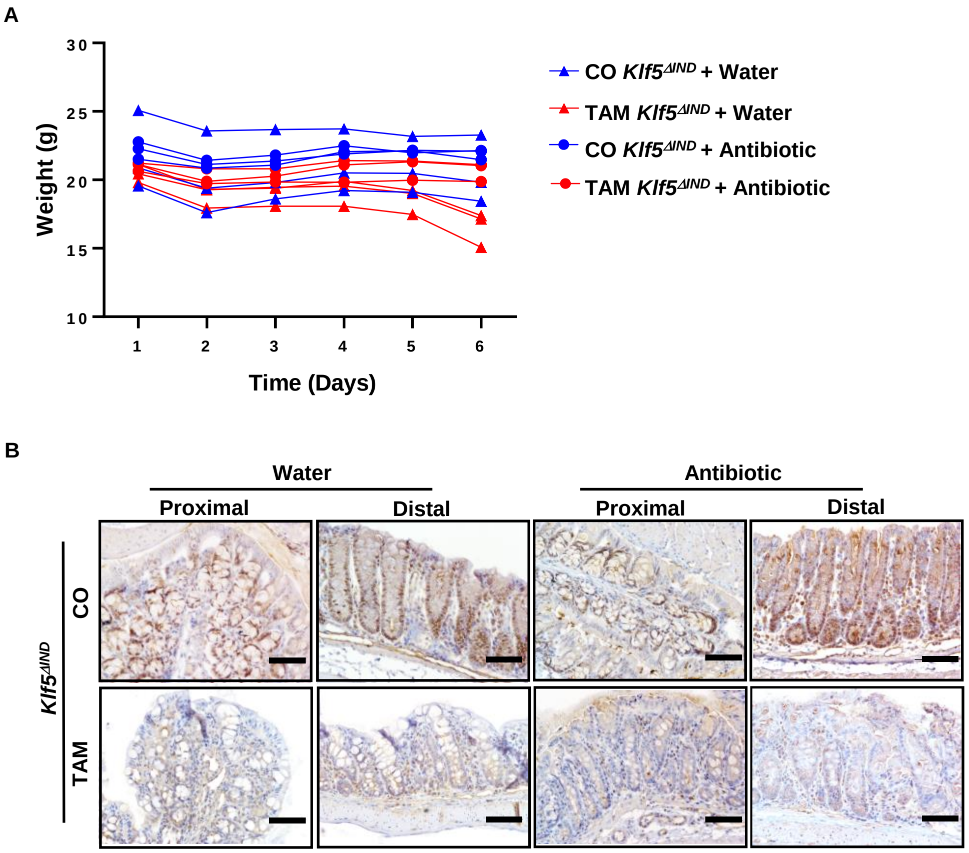

Supplemental Figure 15

Antibodies	Catalog No. and Company	Clone	Fluorophore
CD45	103122, Biolegend	30-F11	APC
Ly6C	128016, Biolegend	HK1.4	FITC
IA/IE (MHCII)	107645, Biolegend	M5/114.15.2	PerCP 5.5
CD11c	117308, Biolegend	N418	PE
Ly6G	127618, Biolegend	1A8	PE/Cy7
F4/80	123131, Biolegend	BM8	BV421
NKp46	137619, Biolegend	29A1.4	BV605
CD11b	101259, Biolegend	M1/70	BV650
FoxP3	15-5773-80, Thermo Fisher	FJK-16s	PECy5
RORgT	12-6981-82, Thermo Fisher	B2D	PE
GATA3	653813, Biolegend	16E10A23	BV421
CD3	100229, Biolegend	17A2	BV650
CD8b	100229, BD	H35-17.2	BV786
CD4	100425, Biolegend	GK1.5	FITC
CD4	100429, Biolegend	GK1.5	AF700
CD4	100426, Biolegend	GK1.5	APC
CD4	100433	GK1.5	PerCP/Cy5.5
CD4	100407, Biolegend	GK1.5	PE
CD4	100409, Biolegend	GK1.5	PE/Cy5
CD4	100421, Biolegend	GK1.5	PE/Cy7
CD4	100437, Biolegend	GK1.5	BV421
CD4	100451, Biolegend	GK1.5	BV605
CD4	100469, Biolegend	GK1.5	BV650
CD4	100453, Biolegend	GK1.5	BV785
CD326	118211, Biolegend	G8.8	AF647
pSTAT3	sc-8059 AF488, Santa Cruz	B-7	FITC
STAT3	678007, VWR	4G4B45	PE

Supplemental Table 1

Antibodies	Catalog No. and Company	Clone	Western Blot	Immunofluorescence/ Immunhistochemistry
Rabbit Anti-STAT3	4904L, Cell Signaling	79D7	1:1000 diluted in 5% Milk	1:100 diluted in 5% BSA w/ TTBS
Rabbit Anti-pSTAT3 (Tyr705)	9145L, Cell Signaling	D3A7	1:1000 diluted in 5% BSA w/ PBS	1:100 diluted in 5% BSA w/ TTBS
Rabbit Anti-KLF5	Ab137676, Abcam	Polyclonal	1:2000 diluted in 5% Milk	1:200 diluted in 5% BSA w/ TTBS
Mouse Anti-GAPDH	CB1001, EMD Millipore	6C5	1:3000 diluted in 5% Milk	N/A
Rabbit Anti-H3	Ab1791, Abcam	Polyclonal	1:3000 diluted in 5% Milk	N/A
Rabbit Anti-IL-17A	Ab79056, Abcam	Polyclonal	N/A	1:100 diluted in 5% BSA w/ TTBS
Rabbit Anti-IL-22	Ab18564, Abcam	Polyclonal	N/A	1:100 diluted in 5% BSA w/ TTBS
Mouse Anti-PanCK	CM011B, Biocare Medical	AE1/AE3	N/A	Prediluted
Rabbit Anti-Ki-67	NB500, Novus Biologicals	Polyclonal	N/A	1:200 diluted in 5% BSA w/ TTBS
Anti-HA	Sc-7392, Santa Cruz	F-7	1:1000 diluted in 5% Milk	N/A
EdU Kit	C10639, Thermo Scientific	N/A	N/A	Kit Manual

Supplemental Table 2
